# Comparison of the functional properties of trimeric and monomeric CaiT of *Escherichia coli*

**DOI:** 10.1038/s41598-019-40516-7

**Published:** 2019-03-07

**Authors:** Susanne Bracher, Daniel Hilger, Kamila Guérin, Yevhen Polyhach, Gunnar Jeschke, Ralph Krafczyk, Giacomo Giacomelli, Heinrich Jung

**Affiliations:** 1 Division of Microbiology, Department of Biology 1, Ludwig Maximilians University Munich, D-82152 Martinsried, Germany; 2 ETH Zurich, Laboratory of Physical. Chemistry, Vladimir-Prelog-Weg 2, Zurich, CH-8093 Switzerland; 3 Present Address: EPFL, Laboratory of Physics of Living Matter LPMV, Rte de la Sorge, Lausanne, CH-1015 Switzerland; 4 Present Address: School of Medicine, Department of Molecular and Cellular Physiology and Medicine, Stanford University, Stanford, CA 94304 USA

## Abstract

Secondary transporters exist as monomers, dimers or higher state oligomers. The significance of the oligomeric state is only partially understood. Here, the significance of the trimeric state of the L-carnitine/γ-butyrobetaine antiporter CaiT of *Escherichia coli* was investigated. Amino acids important for trimer stability were identified and experimentally verified. Among others, CaiT-D288A and -D288R proved to be mostly monomeric in detergent solution and after reconstitution into proteoliposomes, as shown by blue native gel electrophoresis, gel filtration, and determination of intermolecular distances. CaiT-D288A was fully functional with kinetic parameters similar to the trimeric wild-type. Significant differences in amount and stability in the cell membrane between monomeric and trimeric CaiT were not observed. Contrary to trimeric CaiT, addition of substrate had no or only a minor effect on the tryptophan fluorescence of monomeric CaiT. The results suggest that physical contacts between protomers are important for the substrate-induced changes in protein fluorescence and the underlying conformational alterations.

## Introduction

CaiT is a L-carnitine/γ-butyrobetaine antiporter found in the cytoplasmic membrane of *E. coli* and other *Enterobacteriaceae*^1^. The transporter constitutes the start and endpoint of the L-carnitine metabolism in these bacteria. Following uptake, L-carnitine is activated by CoA ester formation and subsequently reduced to γ-butyrobetaine^2–5^. The end product is exported by CaiT in exchange for L-carnitine^1^. The reduction of carnitine stimulates growth of *Enterobacteriaceae* under anaerobic conditions^6^. The metabolic pathway can be used for the enantioselective synthesis of L-carnitine for therapeutic purposes^7,8^.

CaiT belongs to the family of betaine/choline/carnitine transporters (BCCT, TC 2.A.15) of which all members share the capability to transport molecules containing a quaternary ammonium group^9^. Transport follows either a substrate/product antiport mechanism as demonstrated for CaiT^1^, or is driven by an electrochemical H^+^ or Na^+^ gradient as shown for the betaine/Na^+^ symporter BetP of *Corynebacterium glutamicum*^10^. BetP is involved in adaptation of bacteria to osmotic stress and functions not only as transporter but also as a regulator of its own activity^11^.

CaiT forms a trimer in detergent solution as well as in the membrane as deduced from native electrophoresis, gel filtration, cross linking, and electron microscopy^12^. The trimer is confirmed by crystal structures of CaiT^13,14^. Each protomer consists of 12 transmembrane domains (TMDs) out of which TMDs 3 to 12 correspond to a conserved structural core first observed for TMDs 1 to 10 of LeuT^13–15^. The crystal structures of CaiT do not reveal significant conformational differences between the protomers in the trimer^13,14^, and the significance of trimer formation by CaiT is not known. BetP occurs also as a homotrimer^16,17^. Investigation of the role of trimerization in BetP revealed that the trimer is not essential for the ability to accumulate betaine^18,19^. However, BetP loses its function as a regulator when in an engineered monomeric state probably due to altered protein interactions of the extended N- and/or C-terminal domains performing this regulation^18^.

Here, we set out to obtain information on the significance of the oligomeric state of CaiT that despite its structural similarity to BetP is Na^+^-independent and lacks the N- and C-terminal domains involved in regulation of transport activity^1,9^. We generated and verified monomeric variants of CaiT and performed a comparative analysis of properties of these variants and the trimeric wild-type. Specifically, amount and stability of the transporter variants in the membrane, kinetics of transport and the impact of substrate binding on the tryptophan fluorescence of CaiT were analysed. The results show that monomeric CaiT is stable in the membrane and functionally similar to the trimer. Differences in the effect of substrate binding on tryptophan fluorescence between monomeric and trimeric CaiT suggest that a physical contact between the protomers of a trimer has an influence on conformational alterations associated with substrate binding.

## Results

### Disruption of the CaiT trimer

**Figure 1:**
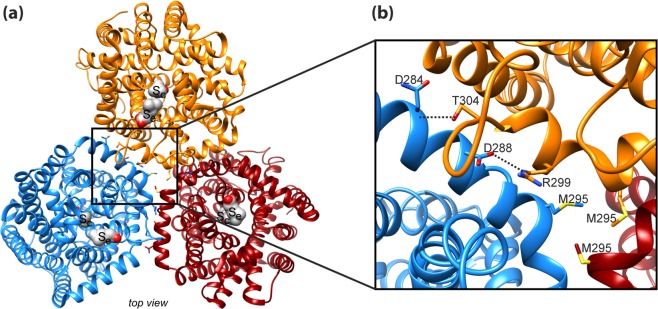
Interactions between the protomers in trimeric CaiT. (**a**) Overview on the structure of trimeric CaiT [PDB# 2WSX^13^] (view from periplasm). The three protomers are colored in orange, red, and blue. Each protomer contains two γ-butyrobetaine molecules (in surface representation) that occupy a central (S_c_) and an external binding site (S_e_). (**b**) Zoom-in view of the structure of CaiT highlighting interactions between the protomers in the trimer on the periplasmic side of CaiT. Tight interactions appear to be mainly stabilized by salt bridges between D288 in helix 7 (periplasmic surface) and R299 in helix 7 of the neighboring protomer as well as by hydrogen bonds between D284 in helix 7 and T304 in loop 7 (connects helix 7 with TMD7) of the neighboring protomer^13^. Furthermore, the side chains of methionine at position 295 close to the kink in helix 7 were found to point to the center of the trimer and are in close proximity. On the cytoplasmic side, the protomers form a large hydrophobic cavity that is probably filled with lipids^13^ (not shown here). Molecular graphics were prepared using the UCSF Chimera package^48^.

Protein-protein interactions stabilizing the trimeric structure of CaiT are observed only on the periplasmic side of the transporter, while the protomers form a large hydrophobic cavity on the cytoplasmic side that is probably filled with membrane lipids^13^. While the long C-terminal domain of BetP is proposed to participate in inter-protomer interaction^16,17^, the respective domain of CaiT is with only two amino acids too short to interact with another protomer. An *in silico* alanine scanning mutagenesis identified amino acids predicted to have high energetic contributions to trimer stability (Table S1). In particular, amino acids of the long, curved α-helix 7 and the periplasmic end of TMD 2 seem to participate in inter-protomer interactions (Table S1 and Fig. S1). In our strategy to destabilize the native trimeric state of CaiT, we focused on the inter-protomer salt bridges between D288 and R299 (helix 7) as well as on the inter-protomer hydrogen bonds between the backbone at position N284 and the side chain of T304 (helix 7) (Fig. 1). In order to interrupt one or more of these interactions, we generated the following amino acid replacements in CaiT: D288A, D288R, D288W, R299A, T304A, and D288A/T304A. In addition, we found that the side chains of M295 at the kink of helix 7 point to each other and are in close proximity (Fig. 1). With the aim to create repulsing forces in the center of the trimer, we introduced a negative charge at this position (M295E).

**Figure 2:**
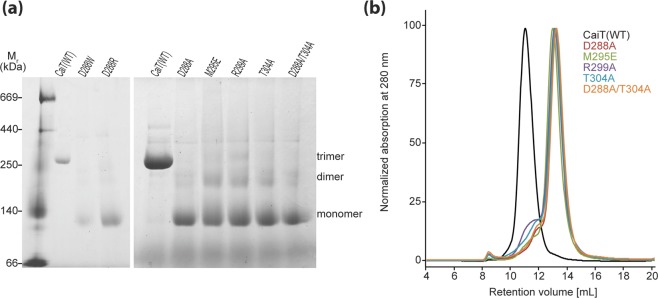
Impact of given amino acid substitutions on the oligomeric state of CaiT in detergent solution. **(a)** BN-PAGE of wild-type and given CaiT variants. Respective genes were expressed in *E. coli*, and the proteins were purified by Ni-NTA affinity chromatography as described^1^. The resulting protein (5 µg per lane) was subjected to BN-PAGE following established protocols^12,40^. Proteins were separated using a 4–16.5% gradient gel. The HMW Native Marker Kit (Amersham Biosciences) was used for molecular weight estimation. **(b)** Gel filtration profile of wild-type and CaiT variants. Proteins (100 µg of each variant solubilized in 0.04% DDM) were analyzed individually with a Superdex 200 10/300 GL column (GE Healthcare). Protein was detected by absorbance measurement at 280 nm.

We tested the effect of the described amino acid substitutions on the oligomeric state of CaiT in detergent solution using blue native-polyacrylamide gel electrophoresis (BN-PAGE) and size exclusion chromatography (SEC). For this purpose, respective genes were expressed in *E. coli*, and the proteins were purified by Ni-NTA affinity chromatography. Analysis by BN-PAGE revealed for all generated CaiT variants that >80% of the protein was in the monomeric state (Fig. 2a). Also, dimer formation was observed, while small amounts of the trimeric transporter were detectable only for CaiT-R299A and CaiT-M295E. The CaiT variants D288A, D288R, and D288W showed in several independent experiments the least tendency to aggregate under the experimental conditions. The SEC analyses revealed that the substitution of D288, M295, R299, and/or T304 yielded monomeric CaiT. Less than 20% of the transporter variants was found in aggregates (Fig. 2b). Here, CaiT-R299A and CaiT-T304A showed the highest tendency to aggregate. In conclusion, best results (no trimer and the least tendency to form unspecific aggregates) were obtained for CaiT containing the substitution D288A or D288R.

### Generation of a functional Cys-free CaiT

**Figure 3:**
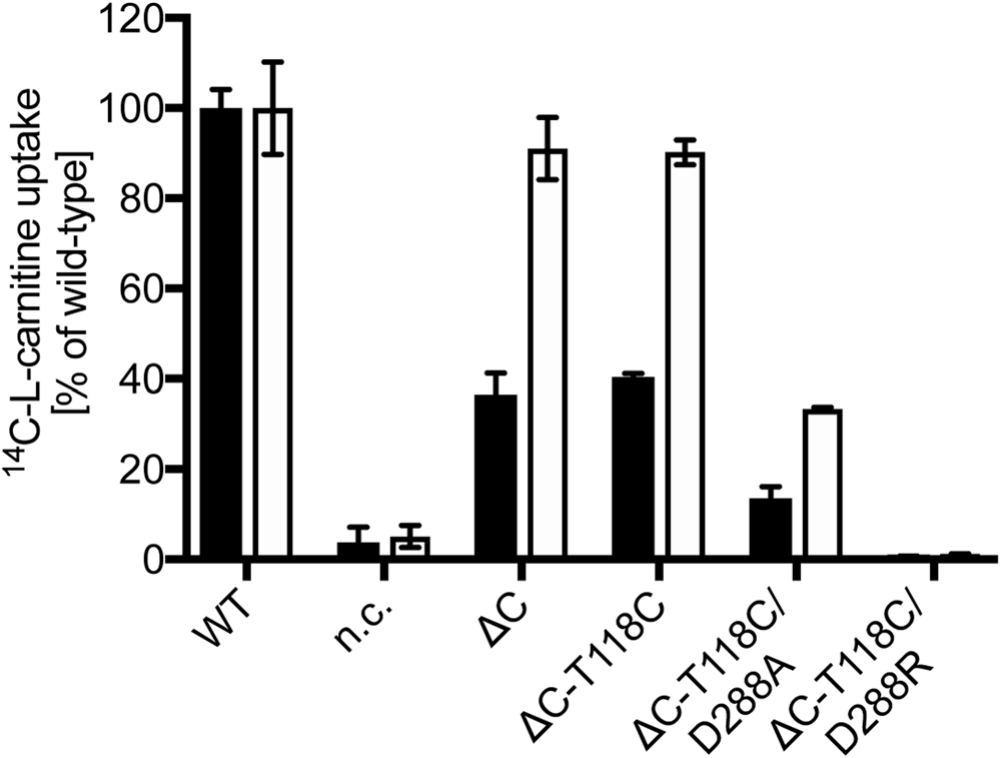
Counterflow activity of CaiT(ΔC) and single Cys CaiT variants. Initial rates of counterflow (black) and maximum accumulation of carnitine in *E. coli* JW0039 (white). Cells were preloaded with 10 mM unlabeled L-carnitine overnight. Aliquots of the cell suspension were then diluted into 400 µL buffer containing 4.5 µM L-[methyl-^14^C]carnitine (55 Ci/mol) resulting in a final carnitine concentration of 54.5 µM. As negative control, cells carrying pET21a (*nc*) were used. Error bars represent standard deviations calculated from independent biological triplicate determinations of the experiment.

To access the oligomeric state of CaiT in the membrane we aimed at using site-directed spin labeling and electron paramagnetic resonance (EPR) spectroscopy. To allow for a site-directed attachment of spin label, we generated a Cys-free CaiT variant (CaiT∆C). For this purpose, all eleven native Cys residues of CaiT were replaced either by alanine or serine depending on the polarity of the environment in the 3D structure. A first CaiT∆C variant (Ala at positions 26, 99, 238, 246, 415, 481; Ser at positions 218, 249, 348, 426, 435) exhibited 3% of the initial uptake rate and 13% of the maximum accumulation of [^14^C]-L-carnitine of the wild-type in L-carnitine counter flow experiments (data not shown). Next, we individually placed each native Cys back into this CaiT∆C variant and analyzed transport activity. Highest activities (>30% of the initial rate of the wild-type) were gained when Cys was placed back at position 99, 246, or 348. In order to improve the functionality of the original CaiT∆C, we tested various combinations of Ala, Ser, and Val at these positions. The CaiT∆C variant with the highest activity (Ala at positions 26, 238, 415, 481; Ser at positions 99, 218, 246, 249, 348, 426, 435) catalyzed counterflow with 32% of the initial uptake rate and 85% of the maximum accumulation of [^14^C]-L-carnitine of the wild-type (Fig. 3). The latter variant was used in all further experiments involving CaiT∆C.

### Assessment of the oligomeric state of CaiT in detergent and in proteoliposomes with distance measurements

**Figure 4:**
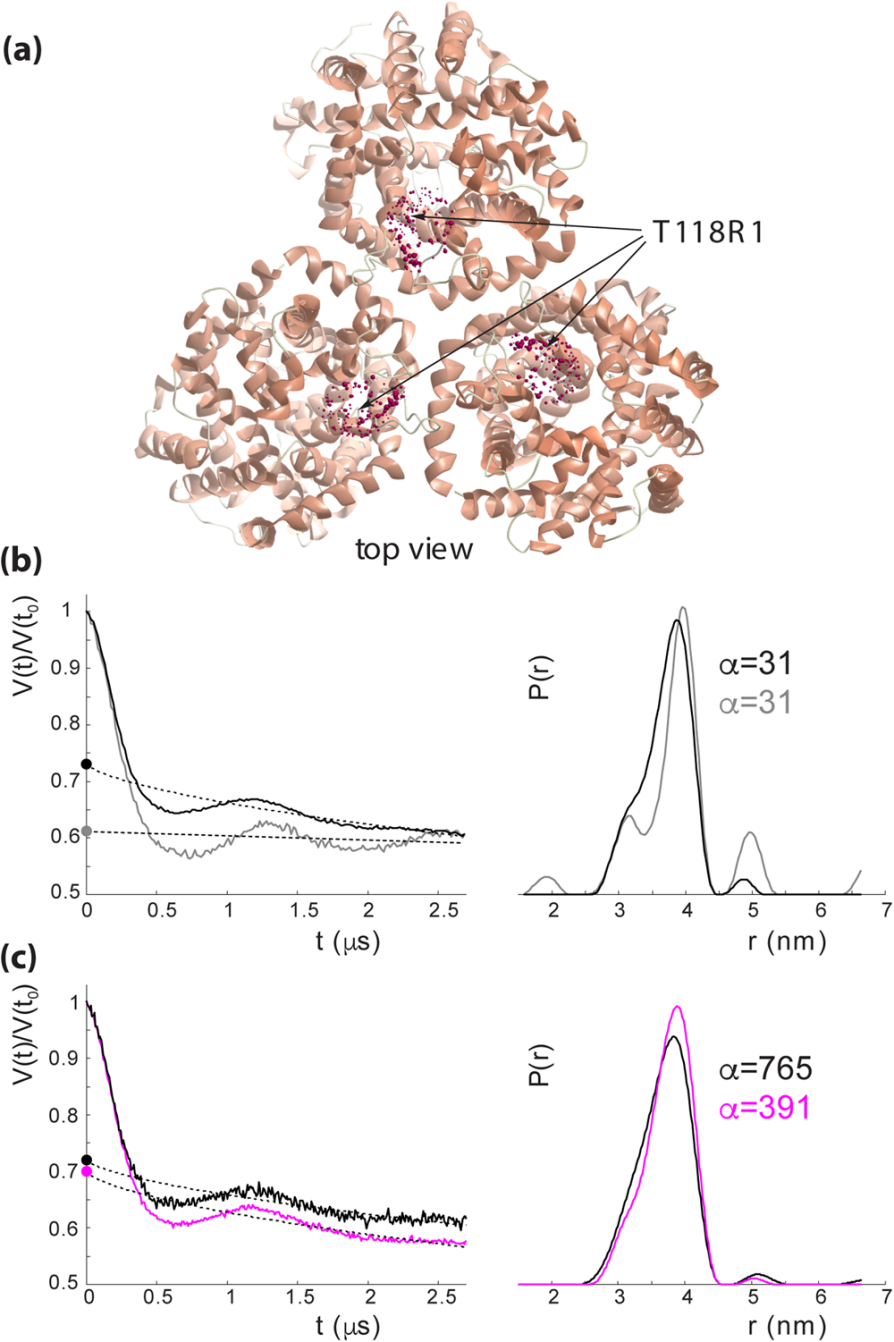
DEER spectroscopy of CaiT(∆C)-R1_118_ in proteoliposomes and in detergent. (**a**) Top view of the CaiT crystal structure (PDB# 2WSX). The native amino acid T118 was substituted by cysteine for subsequent site-directed labeling with (1-oxyl-2,2,5,5-tetramethylpyrroline-3-methyl)-methanethiosulfonate (MTSSL) yielding CaiT(ΔCys)-R1_118._ Red spheres represent relative populations of MTSSL rotamers computed with the rotamer library approach. (**b**) Q-band DEER data of CaiT(ΔC)-R_118_ in proteoliposomes (black) and in the detergent-solubilized state (gray). Left: intensity normalized primary DEER traces. Right: associated distance distributions (probability density functions *P(r)*). **(c)** Effect of carnitine addition on Q-band DEER response of CaiT(ΔC)-R_118_ in proteoliposomes. Black traces – no carnitine added, magenta traces - carnitine added. Left: intensity normalized primary DEER traces. Right: associated distance distributions (probability density functions *P(r)*). Dashed lines in the left panels in (b,c) represent background functions (fits) used for background correction. Total modulation depth (or DEER effect strength) corresponds to a distance between the crossing of the background fits with y-axis (denoted by circles) and 1. The optimal Tikhonov regularization parameter α used during extraction of distance distributions in each case is indicated. Distance distributions are normalized to the area under the *P(r)* curve.

Spin labeling of CaiT was achieved by the reaction of (1-oxyl-2,2,5,5-tetramethylpyrroline-3-methyl)-methanethiosulfonate with Cys placed at the position of T118 (outer end of TMD3) in CaiTΔC yielding CaiTΔC-R1_118_ (R1 represents a nitroxide spin label attached to Cys) (Fig. 4a). This position was chosen because (*i*) substitution has no effect on transport (Fig. 3); (*ii*) *in-silico* simulations of inter-protomer distances between labeled sites with the rotamer library approach^20^, using the crystal structures 2WSX (trimeric CaiT in inward-open conformation) and 3HFX (trimeric CaiT in occluded/outward-open conformation) as templates, yielded moderately broad distance distributions (Fig. S2) supporting the idea that also the experiment may give a well-defined interspin distance. Double Electron Electron Resonance (DEER) measurements of the CaiTΔC-R1_118_ mutant both reconstituted into proteoliposomes and detergent solubilized confirmed these expectations. The Q-band DEER trace acquired on the CaiTΔC-R1_118_ mutant in liposomes shows a clear DEER effect characterized by a moderate total modulation depth of ~0.27. The trace contains two periods of dipolar oscillation in the selected experimental time window corresponding to a compact monomodal distance distribution (Fig. 4b). A very similar result was obtained with the detergent-solubilized samples (Fig. 4b). The only differences were stronger DEER effect (corresponding to a total modulation depth of ~0.39), slightly narrower distance distribution and nearly absent background decay of the primary DEER data. The latter was a result of using non-concentrated detergent-solubilized samples in order to minimize unspecific aggregation effects. Note that the mean of the inter-protomer distance distributions of the CaiTΔC-R1_118_ mutant predicted with the rotamer library approach is smaller while corresponding distribution widths are somewhat larger than the experimentally obtained values (Figs 4 and S3). Binding of L-carnitine to the transporter had essentially no effect on DEER response from the CaiTΔC-R1_118_ mutant except for a slightly larger total modulation depth in the primary DEER data (Figs 4c and S4). These results confirm that CaiT exists as an oligomer (most probably trimer) both in the membrane and in detergent solution.

It is noteworthy, that because CaiTΔC-R1_118_ is a natural oligomer (trimer), the DEER signal from it, in addition to real physical interspin distances, also contains so-called combination frequencies, which do not correspond to any physical distances^21,22^. Simulations performed to assess the significance of such multispin contributions showed that at a total DEER modulation depth of 0.4 (exceeding all experimental values observed) the influence of such contributions on data analysis could safely be neglected (Fig. S2).

**Figure 5:**
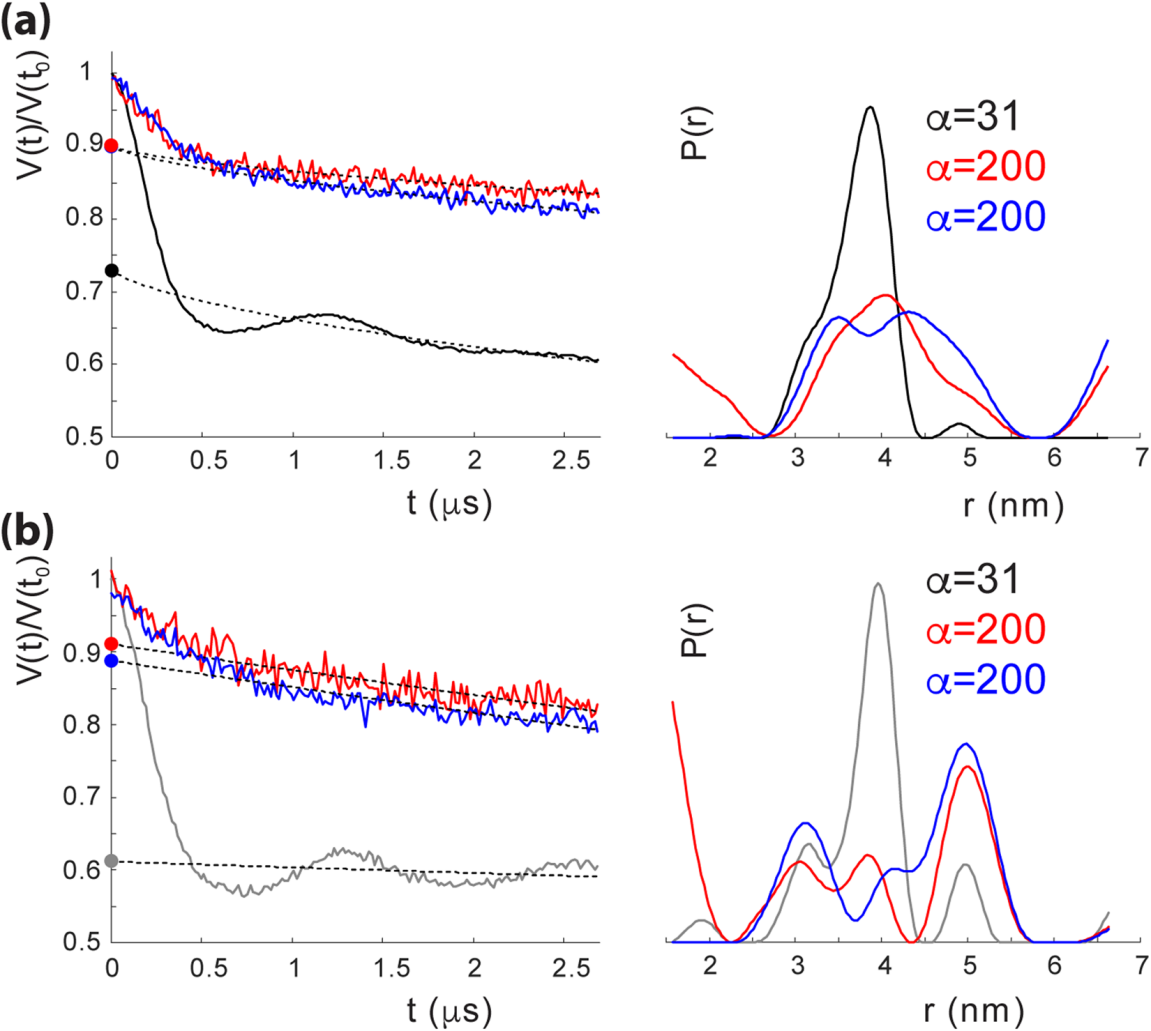
DEER spectroscopy of CaiT(∆C)-D288A, and CaiT(∆C)-D288R in proteoliposomes and in detergent. (**a**) Q-band DEER data of CaiT(∆C)-D288A (red), and CaiT(∆C)-D288R (blue) in proteoliposomes compared to those of CaiT(ΔC)-R_118_ (black). Left: intensity normalized primary DEER traces. Right: associated distance distributions (probability density functions *P(r)*). (**b**) Q-band DEER data of CaiT(∆C)-D288A (red), and CaiT(∆C)-D288R (blue) in detergent compared to those of CaiT(ΔC)-R_118_ (grey). Left: intensity normalized primary DEER traces. Right: associated distance distributions (probability density functions *P(r)*). Dashed lines in the left panels in (**a**,**b**) represent background functions (fits) used for background correction. Total modulation depth corresponds to the difference between intensity 1 of the normalized signal and the crossing of the background fit with y-axis (denoted by circles). The optimal Tikhonov regularization parameter α used during extraction of distance distributions in each case is indicated. Distance distributions are normalized to the area under the *P(r)* curve.

We placed the amino acid substitutions D288A and D288R into CaiTΔC-R1_118_ and subjected the transporters to DEER spectroscopy. The most striking effect of both substitutions was a drastic suppression of the DEER effect in the signal. More precisely, the total modulation depth of the primary DEER trace being a quantitative measure of a number of coupled species (spins) decreased almost 3-fold in case of CaiTΔC-R1_118_ in proteoliposomes and about 4-fold in the detergent-solubilized sample (Fig. 5). In addition, the nature of the DEER signal changed as well: pronounced dipolar oscillations seen before disappeared. Instead, primary DEER data feature a monotonously decaying trace corresponding to a very broad distance distribution in liposomes and in detergent. The origin of that residual weak DEER signal is apparently a nonspecific aggregation dependent on the duration of incubation at room temperature. It is not excluded that the aggregates may contain some fraction of trimers which is difficult to quantify. Incidentally, that fraction seems to be somewhat higher in the D228A and D228R in liposomes than in the detergent when judging from the smaller width of their distance distributions.

These results supplement the BN-PAGE and gel filtration analyses and confirmed that the substitutions D288A or D288R indeed led to the formation of mostly monomers in detergent solution. Importantly, the DEER spectroscopy results indicated that the latter two CaiT variants were mostly monomeric even when reconstituted into proteoliposomes.

### Functionality of monomeric CaiT

Reduction L-carnitine is known to stimulate growth of *E. coli* under anaerobic conditions in the absence of alternative electron acceptors^6^. To test the functionality of CaiT under *in vivo* conditions, *E. coli* JW0039 (*ΔcaiT*) was transformed with plasmid pT7-5/*caiT*(wild-type), pT7-5/*caiT*-D288A, pT7-5/*caiT-*D288R, or pT7-5 without *caiT*, and anaerobic growth in LB medium with and without 50 mM L-carnitine was compared (Fig. S5). Addition of L-carnitine stimulated growth to about the same extent when the cells expressed either *caiT*(wild-type), or *caiT*-D288A. On the contrary, growth was not or only slightly affected by addition of L-carnitine when the cells contained *caiT*-D288R or plasmid pT7-5 without *caiT*. The results were interpreted as a first indication that CaiT-D288A is active similar to the wild type, while transport via CaiT-D288R is impaired.

**Figure 6:**
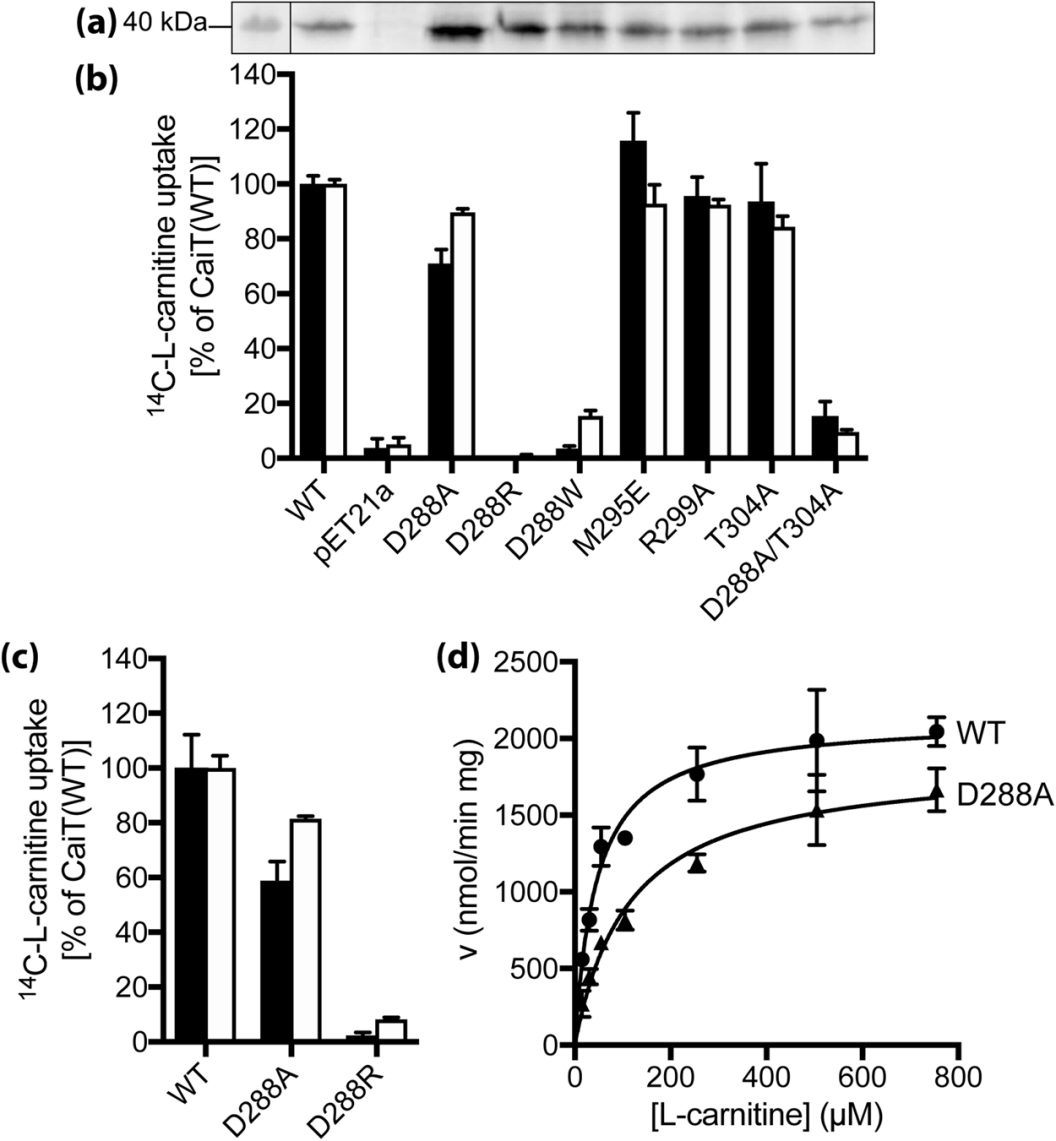
Functional significance of trimeric contacts in CaiT. **(a)** Western blot analysis of CaiT with given amino acid replacements in *E. coli* JW0039 membranes. HRP-linked mouse anti-His IgG was used to detect CaiT. PageRuler Prestained Protein Ladder was used for molecular size estimation. Shown is the section of the blot that contains the CaiT band. The complete gel is presented in Fig. S6. **(b)** Initial rates of counterflow (black) and maximum accumulation of carnitine in *E. coli* JW0039 (white) were determined as described in the legend of Fig. 3. As negative control, cells carrying pET21a (*nc*) were used. **(c)** Initial rates of counterflow (black) and maximum accumulation (white) of carnitine catalyzed by CaiT variants in proteoliposomes. CaiT was purified and reconstituted as described^1^. The lipid to protein ratio of the resulting proteoliposomes was 100:1 (w/w). Proteoliposomes were preloaded with 10 mM unlabeled L-carnitine overnight. Aliquots of the proteoliposome suspension (1–2 µg of protein) were then diluted into 400 µL buffer containing 4.5 µM L-[methyl-^14^C]carnitine (55 Ci/mol) resulting in a final carnitine concentration of 54.5 µM. As negative control, proteoliposomes not preloaded with unlabeled carnitine (*nc*) were used and the obtained transport rates were used for correcting the data. Carnitine counterflow with cells or proteoliposomes was assayed at 25 °C under aerobic conditions using a rapid filtration method as described^1^. **(d)** Michaelis-Menten kinetics of L-carnitine counterflow in proteoliposomes reconstituted with wild-type CaiT (WT) or CaiT-D288A. Initial rates of L-[methyl-^14^C]carnitine uptake were measured at given substrate concentrations and plotted using the kinetic module of GraphPad Prism. For all experiments, standard deviations were calculated from triplicate determinations of a representative experiment.

Next, the functionality of CaiT was analyzed in L-carnitine counterflow experiments^1^. For standard test conditions, cells or proteoliposomes were preloaded with 10 mM L-carnitine, and the initial external L-[^14^C]-carnitine concentration was adjusted to 54.5 µM. While all amino acid substitutions analyzed in this investigation seem to destabilize the trimer, the impact on counterflow activity was diverse. Cells of *E. coli* with CaiT-D288A catalyzed L-[^14^C]-carnitine uptake into cells (initial rate about 75% of the wild-type) while cells with CaiT-D288R were unable to transport L-[^14^C]-carnitine (Fig. 6b). Importantly, both CaiT variants were present in the membrane in comparable amounts (Fig. 6a). Similarly, purified and reconstituted CaiT-D288A was highly active (initial rate about 60% of the wild-type), and CaiT-D288R did again not show significant activity (Fig. 6c).

For a more detailed kinetic analysis, initial rates of counterflow were determined with CaiT-containing proteoliposomes at different external concentrations of L-carnitine (Fig. 6d). Determination of the Michaelis-Menten parameters revealed an about 2fold higher *k*_*m(L-carnitine)*_ of CaiT-D288A compared to the wild-type. The maximum rate of transport (*V*_*max*_) of the CaiT variant reached 87% of the corresponding wild-type value (Table 1).

**Table 1:** Kinetic parameters of carnitine counterflow of CaiT bearing substitutions of given amino acids.

CaiT variant	*K*_*m*_ [µM]	*V*_*max*_ [µmol min^−1^ mg^−1^]
wild-type	45.3 ± 6.9	2.13 ± 0.08
D288A	114.1 ± 17.9	1.86 ± 0.09
D288R	not detectable	<3% of wild-type

Carnitine counterflow was measured with proteoliposomes reconstituted with purified CaiT (lipid to protein ratio of 100:1 (w/w)) as described^1^. The proteoliposomes were preloaded with 10 mM unlabeled L-carnitine at 4 °C overnight. Counterflow was initiated by 200 to 800fold dilution of preloaded proteoliposomes into buffer containing L-[methyl-^14^C]carnitine at final concentrations ranging from 0.014 to 1 mM at 25 °C. Proteoliposomes not preloaded with L-carnitine served as negative control, and resulting transport rates were used for correction. The data were fitted to the Michaelis-Menten equation and replotted according to Lineweaver-Burk and Eadie-Hofstee using the kinetic module of the SigmaPlot software or GraphPad Prism. Shown are mean values and standard deviations calculated from three independent experiments.

Finally, out of the other CaiT variants initially shown to be monomeric in detergent solution, CaiT-M295E, -R299A, and -T304A were as active as the wild-type, while CaiT-D288W and D288A/T304A showed activities of 4 and 18% of the wild-type in cells (Fig. 6a,b).

Taken together, these results indicate that monomeric CaiT is sufficient to form a functional translocation pathway (e.g. CaiT-D288A). The reduced activities of CaiT-D288W and -D288A/T304A, and the inactivity of the D288R variant may be explained by altered interactions of the transporter with the phospholipid bilayer leading to an inhibition of the transport cycle.

### Tryptophan fluorescence

**Figure 7:**
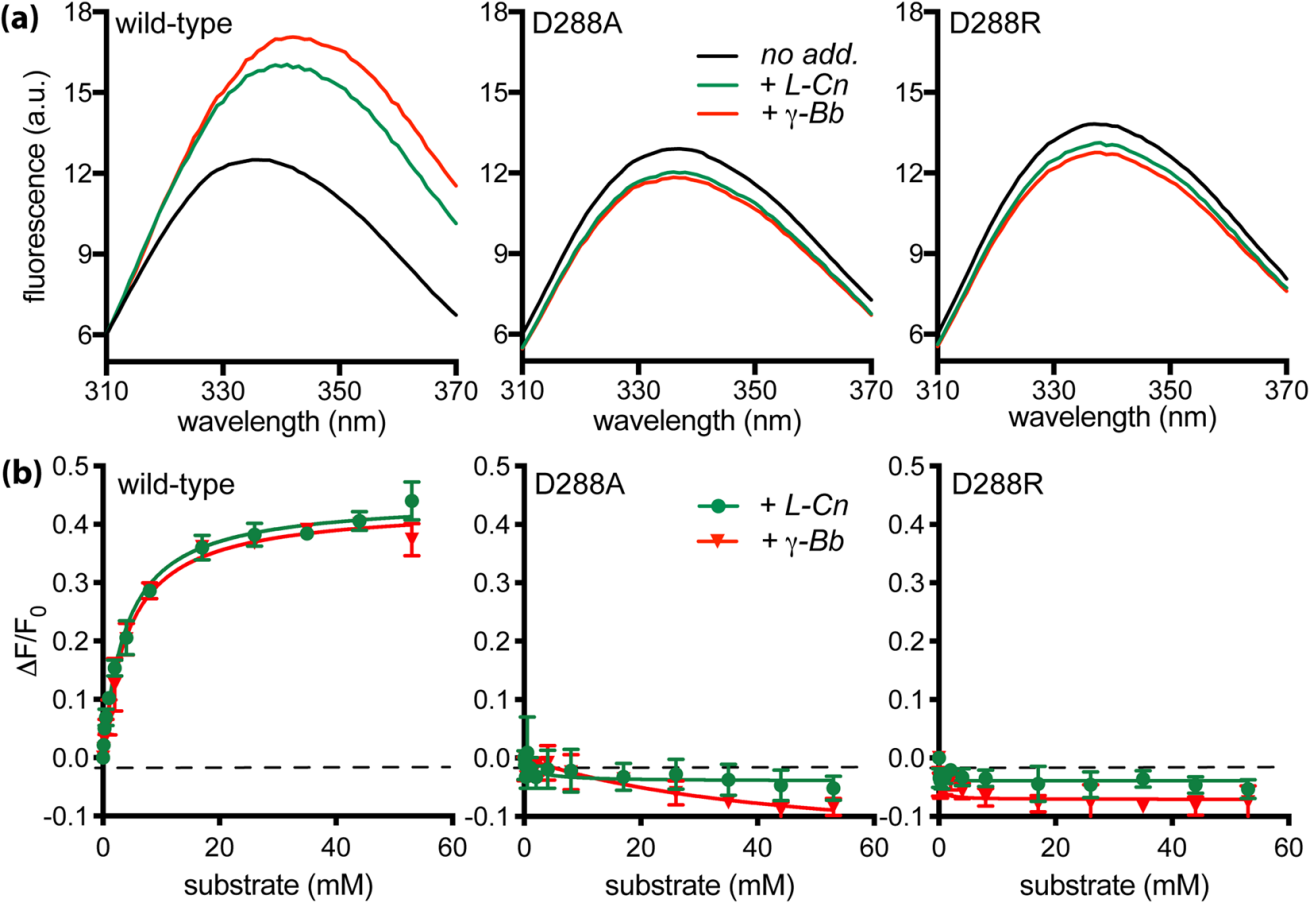
Impact of substrate binding on the tryptophan fluorescence of trimeric and monomeric CaiT in the solubilized state. **(a)** Fluorescence emission spectra were recorded between 310 and 370 nm with an excitation wavelength of 295 nm. Purified CaiT (80 μg mL^−1^) was solubilized in 100 mM potassium phosphate buffer, pH7.5, 2 mM β-mercaptoethanol, 10% glycerol, 200 mM imidazole, 0.04% *n*-dodecyl-*β*-D-maltoside. All fluorescence data were corrected for a buffer blank and for dilution effects. Shown are the emission spectra of CaiT without substrate (no add.), with 53 mM L-carnitine (+L-Cn) or γ-butyrobetaine (+γ-Bb). (**b**) L-carnitine or γ-butyrobetaine was added stepwise to final concentrations between 0 to 53 mM. The resulting change of the fluorescence intensity was measured at 338 nm and plotted using the kinetic module of the software GraphPad Prism. Shown are mean values and standard deviations calculated from three experiments.

**Figure 8:**
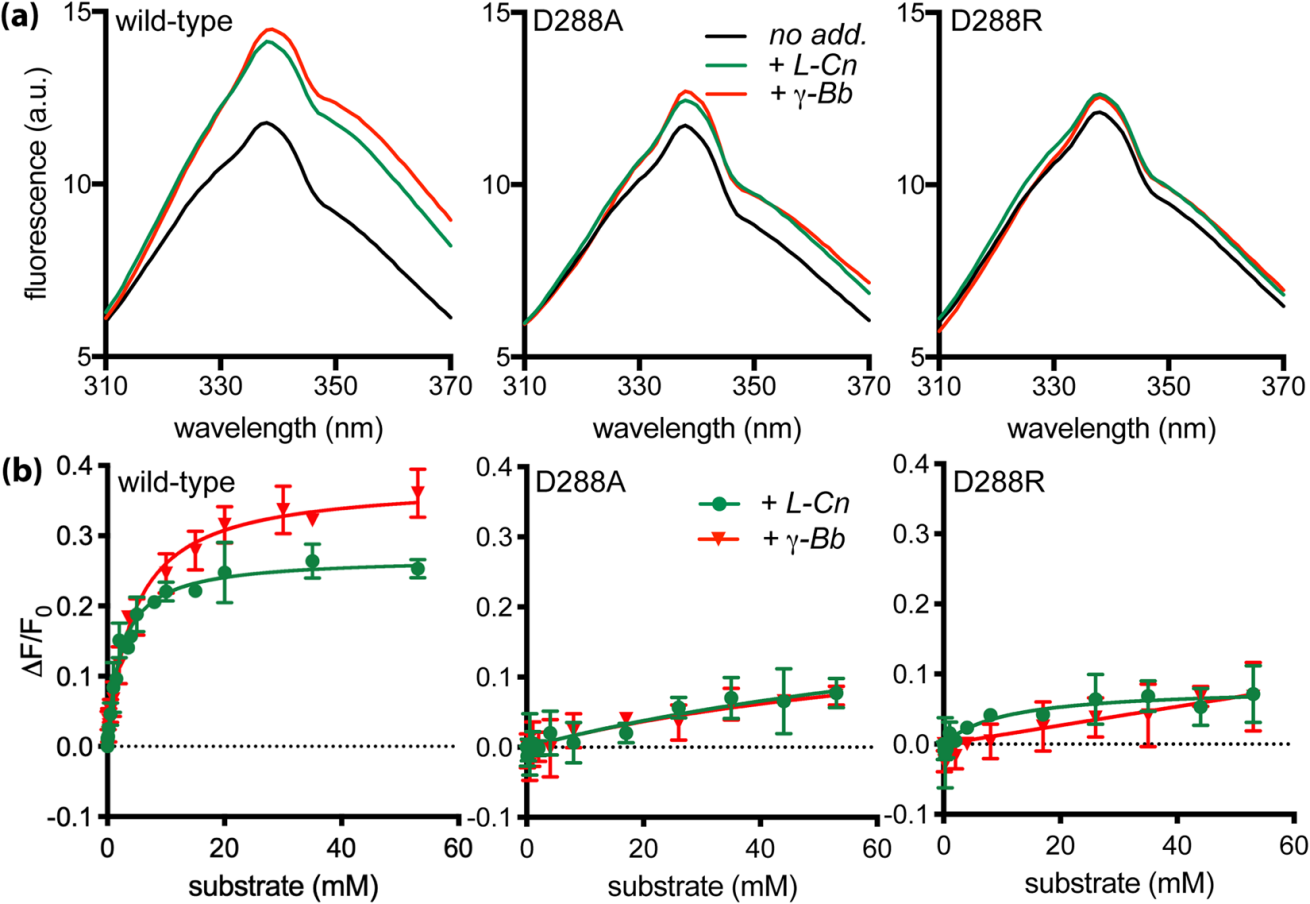
Impact of substrate binding on the tryptophan fluorescence of trimeric and monomeric CaiT reconstituted into proteoliposomes. **(a)** Fluorescence emission spectra of given CaiT variants in proteoliposomes [80 μg mL^−1^, lipid to protein ratio 20:1 (w/w)] in 100 mM potassium phosphate buffer, pH8, 2 mM β-mercaptoethanol, 5 mM MgCl_2_ at 25 °C. All fluorescence data were corrected for a buffer blank and for dilution effects. Shown are the emission spectra of CaiT without substrate (no add.), with 53 mM L-carnitine (+L-Cn) or γ-butyrobetaine (+γ-Bb). (**b**) Substrate-dependent change of the fluorescence intensity. All other conditions were as described in the legend of Fig. 7.

Substrate binding to solubilized wild-type CaiT increased the intensity of tryptophan fluorescence and induced a red shift of the emission maximum^1^ (Fig. 7). Titration of the effect on fluorescence intensity with the solubilized wild-type protein yielded *K*_*d*_ values of 3.3 mM and 3.4 mM for L-carnitine and γ-butyrobetaine, respectively. The values for the reconstituted CaiT were in the same order of magnitude (Table 2). Performance of the same experiments with CaiT containing the substitutions D288A or D288R revealed significant changes of impact of substrate binding on tryptophan fluorescence. Contrary to the wild-type, the fluorescence of the solubilized monomeric protein variants was not significantly affected by the addition of L-carnitine or γ-butyrobetaine. Also, a red shift of the emission maximum was not observed (Table 2, Fig. 7a). Of note, all values were corrected for the effect of buffer without substrate. Trials to determine *K*_*d*_ values for the solubilized CaiT variants failed due to the lack of significant effects of substrate on the fluorescence. For the reconstituted monomeric CaiT variants, addition of L-carnitine or γ-butyrobetaine caused only a slight increase of the fluorescence intensity (Table 2 and Fig. 8). Nonetheless, titration of substrate to the latter reconstituted CaiT variants yielded *K*_*d*_ values similar to the wild-type (Table 2). These results suggest that the oligomeric state of CaiT has a strong impact on conformational alterations underlying substrate-induced changes of the tryptophan fluorescence.

**Table 2:** Parameters of substrate binding to CaiT bearing given substitutions determined by tryptophan fluorescence analyses.

CaiT variant	L-carnitine			γ-butyrobetaine		
	K_d_ [mM]	max ΔF/F_o_^a^	λ_max_ [nm] (Δλ_max_)	K_d_ [mM]	max ΔF/F_o_	λ_max_ [nm] (Δλ_max_)
**solubilized CaiT**						
wild-type	3.3 ± 0.2	0.43 ± 0.02	336 (+5)	3.4 ± 0.1	0.42 ± 0.01	336 (+6)
D288A	not detectable	−0.05 ± 0.01	337 (−1)	not detectable	−0.18 ± 0.04	337 (−1)
D288R	not detectable	−0.04 ± 0.01	337 (+1)	not detectable	−0.07 ± 0.01	337 (+1)
**CaiT reconstituted into proteoliposomes**						
wild-type	2.4 ± 0.2	0.27 ± 0.01	338 (0)	4.5 ± 0.5	0.38 ± 0.01	338 (+1)
D288A	1.8 ± 0.9	0.08 ± 0.01	338 (0)	3.3 ± 1.3	0.08 ± 0.01	338 (0)
D288R	1.9 ± 1.0	0.07 ± 0.01	338 (0)	11.4 ± 4.4	0.11 ± 0.01	338 (0)

^a^*max ΔF/F*_*o*_ represents a saturation value of a hyperbolic fit and was determined using the kinetic module of the software GraphPad Prism.

Binding of L-carnitine and γ-butyrobetaine was measured with solubilized CaiT and with CaiT reconstituted into proteoliposomes (lipid to protein ratio of 100:1 (w/w)) as described^1^. Experiments were performed as described in the legends of Figs 7 and 8. The change of the fluorescence intensity measured at 338 nm was plotted using the kinetic module of the software GraphPad Prism. Shown are mean values and standard deviations calculated from three experiments.

### Stability of monomeric CaiT

**Figure 9:**
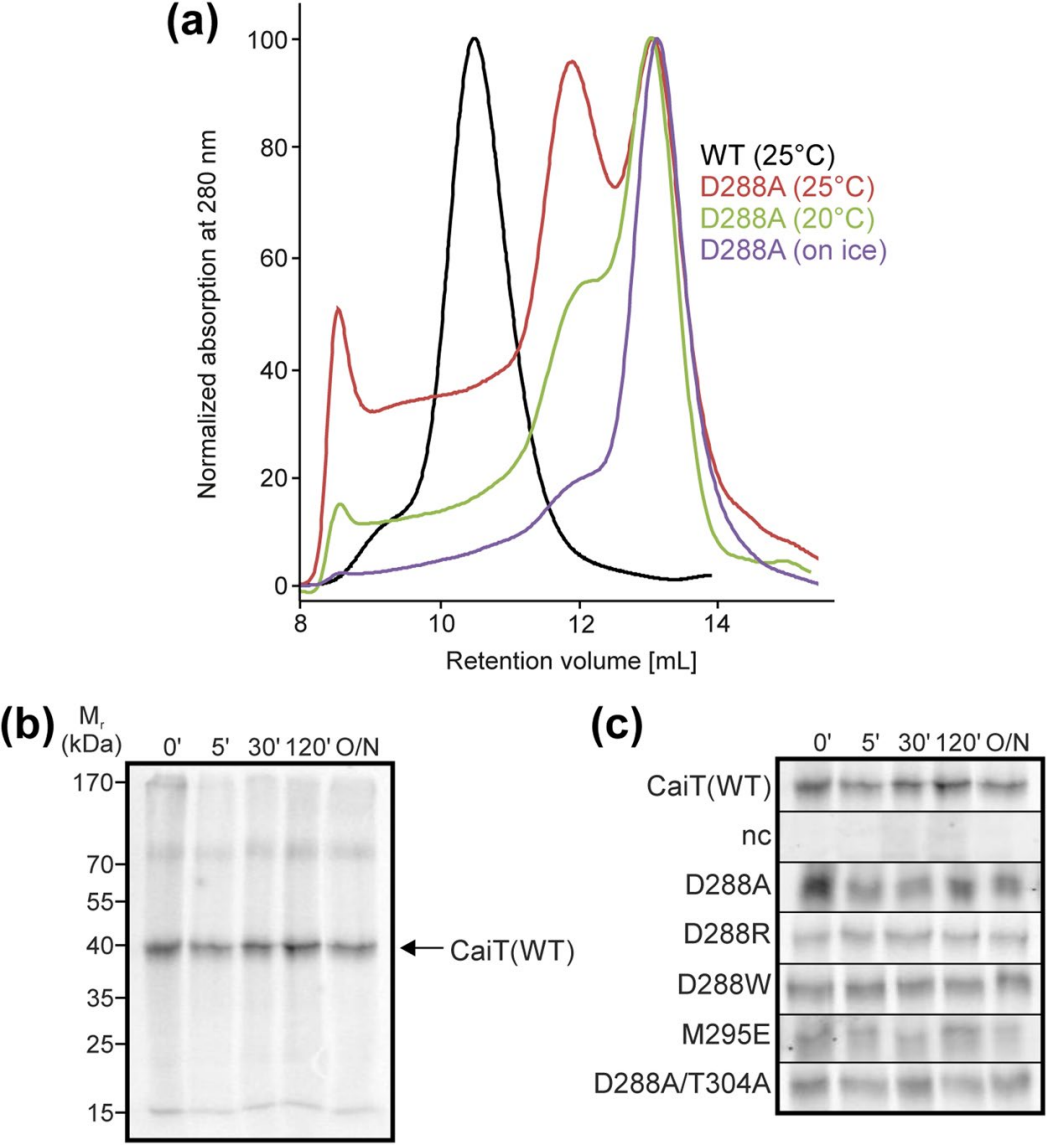
Influence of the oligomeric state on the stability of CaiT. (**a**) The stability of the purified CaiT variants in the solubilized state (100 mM potassium phosphate buffer pH7.5, 2 mM β-mercaptoethanol, 0.04% (w/v) dodecyl β-D-maltoside) was analyzed by incubation of the proteins at given temperature for 60 min and gel filtration (Superdex 10/300 GL column) similar as described for Fig. 2c. (**b**) The stability of CaiT(WT) in *E. coli* was tested in a pulse-chase experiment. Cells were incubated with [^35^S]methionine at 30 °C for 10 min before removal of the zero-time point aliquot and addition of an excess of unlabeled methionine (2 mg/mL final concentration). Further aliquots were taken at given time points of incubation. Cells were disrupted by sonication, membranes were prepared and subjected to SDS-PAGE (12.5%). Radioactivity was detected with a Phosphor imager after drying the gel. The arrow indicates the position of CaiT. (**c**) Stability of CaiT(WT) and monomeric CaiT variants. The experiment was performed as described for (**b**). The sections of the gels containing CaiT are shown. The complete gels are presented in Fig. S7.

Trimeric CaiT is relatively stable in detergent solution even at room temperature, while solubilized monomeric CaiT has a high tendency to form non-specific aggregates (Fig. 9a). However, when incorporated into the cytoplasmic membrane, all monomeric CaiT variants were as stable as the wild-type as indicated by pulse-chase experiments (Fig. 9b,c).

## Discussion

CaiT was previously shown to form a stable trimer both in detergent solution and in the membrane^12–14^. Starting from the crystal structures of CaiT^13,14^, we have identified and experimentally verified amino acids crucial for trimer stability. Amino acid substitutions leading to interruption of the inter-protomer salt bridge between D288 and R299 (helix 7) or of the inter-protomer hydrogen bond between N284 (peptide backbone) and T304 (helix 7) destabilize the trimer and lead to monomeric CaiT in detergent solution. Monomeric CaiT is observed also upon introduction of negative charges (M295E in helix 7) in the center of the interfaces of the three protomers. Multiple substitutions (e.g., D288A/T304A) lead with respect to monomer formation to similar results as the single substitutions. DEER analysis of CaiTΔC-R1_118_ in detergent solution and in proteoliposomes yields a well-defined inter-protomer distance as expected for the trimer. These results indicate that neither native Cys residues nor the C-terminal 6His tag interfere with trimer formation. Contrary to CaiTΔC-R1_118_, low modulation depths and broad distance distributions are found in the corresponding CaiT variants D288A and D288R in both environments suggesting that a minor fraction of otherwise monomeric protein forms disordered aggregates. Neither the salt bridge (D288, R299) nor M295 are conserved in members of the BCCT family (Fig. S1). Only the hydrogen bond (T304, backbone N284) appears to be present in all family members. Substitution of the corresponding threonine in BetP (T351A) leads to partly dimeric protein. Contrary to CaiT, a second substitution (e.g., W101A) is necessary to generate mostly monomeric BetP^18^. The discrepancy could be due to the lack of other structural elements in CaiT (e.g., the extended cytoplasmic N- and/or C-terminal domains present in BetP) that may contingently contribute to protomer interactions [albeit the C-terminal domain of BetP is not essential for trimer formation^18^]. In any case, these previous results on BetP^18^ and our results with CaiT confirm that periplasmic helix 7 is crucial for trimer formation by BCCT proteins.

The thermodynamic basis of monomer formation by one or two amino acid substitutions remains enigmatic. For CaiT-D288A we can only speculate that repulsive forces originating from the positive charge remaining at position R299 and/or favorable interactions of the side chain with phospholipid head groups on the periplasmic side prevent trimer formation. Steric disruptions of the contact surface’s shape complementarity and an enhanced affinity of this surface for the lipid bilayer were previously discussed as mechanisms to inhibit dimer (oligomer) formation of integral membrane proteins^23^. In the given example, introduction of two tryptophan residues at TMD ends caused monomerization of the dimeric H^+^/Cl^−^ exchanger ClC-Ec1^23^.

Functional analyses reveal that the CaiT protomer constitutes a full functional unit. Although our DEER measurements cannot 100% rule out that a minor fraction of the transporter is in a trimeric state, *V*_*max*_ values of CaiT-D288A in proteoliposomes of 87% of the wild-type indicate that monomeric CaiT can catalyze L-carnitine transport. Our results are in principle in agreement with previous investigations showing that monomeric versions of BetP^18^, ClC-Ec1^23^ and H^+^/Na^+^ antiporter NhaA^24^ are able to catalyze complete transport processes.

Oligomerization does not seem to be bound to specific structural or functional classes of proteins. In fact, transporters even with similar structural folds exist as monomers [LeuT^15^] or trimers [CaiT^13^, BetP^18^], and function as solute/Na^+^ symporters [LeuT^15^, BetP^10^] or substrate antiporter [CaiT^1^]. What is then the functional role of the oligomerization of secondary transporters? The significance is obvious for small transporters like, for example, EmrE (contains four TMDs), for which a homodimer is required to form the translocation pathway^25^. For larger transporters with eight and more TMDs, in which each protomer is thought to represent a functional unit, oligomerization is often assumed to have a structure-stabilizing effect^18,26^. Supporting this idea, functional comparison of monomeric and dimeric NhaA indicates that dimerization is crucial for the stability of the antiporter under extreme pH and salt stress conditions^24^. For CaiT, the stability aspect does not seem to apply since amounts and stability of monomeric and trimeric CaiT in the membrane are comparable. Only the tendency to aggregate in detergent solution differs significantly between the two states of CaiT.

Furthermore, the structural asymmetry seen in some crystal structures of trimeric transporters [e.g., ArcB^27^, BetP^28^] suggests functional interactions (e.g., cooperativity) between the protomers. Additional experimental evidence for this hypothesis is scarce. Support comes from the analysis of cross-linked AcrB showing that inactivation of only one of the three protomers inactivates the entire complex^29^. Functional complementation of defective protomers of the plant ammonium transporter AMT are also seen as indication for functional interactions between the protomers^30^. On the contrary, cross-linking of the protomer interfaces in the trimeric glutamate transporter GltT does not affect transport suggesting that the interfaces are rigid during the transport cycle^31^. Furthermore, analysis of monomeric BetP revealed that the transporter is no longer regulated by osmotic stress, but retains its ability to transport betaine^18^. However, a heterotrimer approach provides evidence for the absence of functionally significant conformational crosstalk between the protomers of BetP on the level of both transport and regulation^19^. Nonetheless, the difference between the activities of monomeric BetP (13% of the *V*_*max*_ of the trimer) and the orphan protomer in the heterotrimer (full activity) suggests that optimal activity requires physical contacts between the protomers^18,19^.

Does our investigation on CaiT add new aspects for the meaning of oligomerization of BCCT proteins? Different from the results on BetP, monomeric CaiT (CaiT-D288A) is almost as active as the trimer (87% of the *V*_*max*_). Consequently, physical contacts between the protomers do not seem to be as important for CaiT as for BetP. It is tempting to speculate that the additional regulatory domains of BetP and their interactions with adjacent (active or inactive) protomers are responsible for this difference. This idea is challenged by the finding that alteration of the “docking site” at loop 2 of the regulatory C-terminal domain of BetP in the adjacent inactive protomer (heterotrimer approach) does not affect regulation of the active protomer^19^. However, there may be other inter-protomer interactions of the C-terminal and the less explored N-terminal domains of BetP [cp.^32^] that do not occur in CaiT.

The most prominent difference between monomeric and trimeric CaiT is revealed by analyses of the protein (tryptophan) fluorescence. While substrate binding to solubilized or reconstituted trimeric CaiT causes an increase of the fluorescence intensity and a red shift of the emission maximum^1^, this effect is highly reduced for monomeric CaiT in proteoliposomes and absent in detergent solution (this investigation). This observation is surprising since the conformational alterations underlying the change in tryptophan fluorescence are thought to be part of the transport cycle^1,13^. In fact, comparison of the crystal structures of CaiT with and without bound substrate leads to W323 as the only tryptophan that changes its environment. Therefore, re-orientation of W323 is seen as cause of the observed change of tryptophan fluorescence of the trimer^13^. Re-orientation of W323 is considered essential for substrate binding as its side chain blocks the central substrate binding site in the absence of substrate^13^. We have the following suggestions to explain the absence of the change in fluorescence in highly active monomeric CaiT: (*i*) The substitution necessary for generation of the monomer (e.g., D288A) keeps W323 in an “open position” also in the absence of substrate. However, the identical fluorescence emission spectra of monomeric and trimeric CaiT without substrate argue against this idea. (*ii*) Re-orientation of W323 is not the cause for the substrate-dependent change of fluorescence or at least not the only one. If true or not, one would expect that inhibition of any intra-protomer re-arrangement that is coupled to substrate binding affects function. However, the similar kinetic parameters of transport argue against this explanation. (*iii*) The fluorescence effect is the result of inter-protomer interactions that are unimportant for the function of the monomer. While this appears to be the simplest explanation, the crystal structures of CaiT^13^ do not reveal any asymmetry. In conclusion, while a physical contact between the protomers appears to be crucial for the conformational alterations underlying the substrate-induced change of the tryptophan fluorescence, none of the above explanations can currently be ruled out.

Taken together, our results show that the protomers of trimeric CaiT represent fully functional units that as monomers catalyze transport with kinetic parameters close to wild-type. While inter-protomer interactions appear to be unimportant for transport, the physical contact between the promoter may facilitate conformational alterations associated with substrate binding as suggested by the different outcomes of the fluorescence analyses of monomeric and trimeric CaiT. In future, the heterotrimer approach and more detailed comparative analyses of conformational alterations in monomeric and trimeric CaiT may provide further clues for the meaning of the oligomerization of transporters.

## Materials and Methods

### Bacterial strains and plasmids

*E. coli* DH5α [F^−^ ϕ80d *lacZ* ΔM15 Δ(*lac*ZYA-*argF*) U169 *deoR recA1 endA1 hsd* R17(r_k_−, m_k_+) *phoA supE44* λ^−^ *thi-1 gyrA96 relA1*] was used as carrier for plasmids. *E. coli* JW0039 (F^−^ *ΔcaiT753::kan Δ*(*araD-araB*)*567 ΔlacZ4787*(::rrnB-3) *λ*^−^ *rph-1 Δ*(*rhaD-rhaB*)*568 hsdR514*)^33^ or *E. coli* BL21(DE3) pLysS (F^−^ *ompT hasdSB* (rB^−^ mB^−^) *gal dcm* (DE3) pLysS (cam^R^)) (Novagen) harboring given plasmids was used for expression of the *caiT* gene *of E. coli* and biochemical assays. For the pulse-chase experiments, *E. coli* WG170 (F^−^ *trp lacZ rpsL thi Δ*(*putPA*)*101 proP219*)^34^ was used. Derivatives of pT7-5^35^ containing *caiT* under control of the *lac* promoter/operator for expression (pT7-5/caiT)^1^ were used for immunological analysis and pulse-chase experiments. For overexpression, *caiT* was cloned into plasmid pET21a (Novagen).

### Site-directed mutagenesis

Desired nucleotide substitutions in *caiT* were generated by PCR with Phusion DNA polymerase using the plasmids described above as a template and synthetic mutagenic oligonucleotides following the QuikChange Site-Directed Mutagenesis protocol developed by Stratagene (La Jolla, CA). The plasmid DNA was verified by sequencing using an ABI 3730 capillary sequencer.

### Immunological Analysis

*E. coli* JW0039 harboring plasmids pT7-5/*caiT* encoding CaiT with given amino acid replacements were grown aerobically in LB medium^36^ containing 100 μg/mL ampicillin at 37 °C. Overnight cultures were diluted 10-fold and were allowed to grow to an optical density at 420 nm (OD420) of 1.0, followed by induction with 0.5 mM IPTG for 2 h. Cells were harvested by centrifugation at 16,100 *g* for 10 min and washed with 100 mM potassium phosphate buffer, pH7.5, 2 mM β-mercaptoethanol at 4 °C. Cells were disrupted by sonification, and randomly oriented membrane vesicles were prepared as described^37^. Relative amounts of CaiT with given amino acid replacements in membranes of *E. coli* JW0039 harboring plasmid pT7-5/*caiT* were estimated by Western blot analysis with HRP-linked mouse monoclonal IgG His-probe Antibody (H-3) (Santa Cruz Biotechnology) directed against the His tag at the C terminus of each CaiT variant by the enhances chemiluminescence method according to the manufacturer’s protocol. Membrane vesicles of *E. coli* JW0039 harboring pT7-5 served as negative control.

### Purification of CaiT

*E. coli* JW0039 harboring plasmids pET21a/*caiT* encoding CaiT with given amino acid replacements were grown aerobically in LB medium and induced with 0.5 mM IPTG as described above. Randomly oriented membrane vesicles were prepared using a high-pressure cell disruption system (Constant Systems) at 1.35 kbar as described^37^. CaiT variants were purified via the C-terminal His tag using a Ni-NTA agarose essentially as described^1^.

### Reconstitution of CaiT

Purified CaiT was reconstituted into liposomes as described^1^. A lipid to protein ratio of 100:1 (w/w) was used for transport assays, a ratio of 40:1 (w/w) was adjusted for Double Electron Electron Resonance (DEER) spectroscopy, and a ratio of 20:1 (w/w) was used for fluorescence measurements.

### Transport assay

*E. coli* JW0039 harboring plasmids pET21a/*caiT* encoding CaiT with given amino acid replacements were grown aerobically in LB medium as described above. Overnight cultures were diluted 10-fold and were allowed to grow to an optical density at 420 nm (OD_420_) of 1.0, followed by further growth for 2 h. Cells were harvested by centrifugation at 16,100 *g* for 10 min and washed with 100 mM potassium phosphate buffer, pH7.5, 2 mM β-mercaptoethanol at 4 °C. Cells were resuspended in the same buffer and adjusted to OD_420_ = 100 and preloaded with 10 mM unlabeled L-carnitine overnight at 4 °C. 2 µL aliquots of the cell suspension were then diluted into 400 µL of buffer containing 4.5 µM L-[methyl-^14^C]carnitine (55 Ci/mol) resulting in a final L-carnitine concentration of 54.5 µM. L-Carnitine counterflow was assayed at 25 °C under aerobic conditions. Transport was terminated at the desired timepoints by addition of ice cold 100 mM KH_2_PO_4_, 100 mM LiCl, pH 6.0 and rapid filtration as described^1^. Initial rates of transport were calculated from the initial linear portion of the time course, and steady-state levels of L-[methyl-^14^C]carnitine accumulation were taken from time points after leveling off of the uptake curve. Standard deviations were calculated from at least triplicate determinations. *E. coli* JW0039 harboring pET21a served as negative control. For determination of K_*m*(Carnitine)_, CaiT-containing proteoliposomes were used. For reconstitution into liposomes, a lipid to protein ratio of 100:1 (w/w) was adjusted. Proteoliposomes were preloaded with 10 mM unlabeled L-carnitine at 4 °C overnight. Proteoliposomes were extruded (400 nm) and concentrated by centrifugation to a protein concentration of 1–2 mg/mL. Aliquots of the proteoliposome suspension (1–2 µg of protein) were diluted 200- or 400-fold into 100 mM potassium phosphate buffer, pH7.5, 2 mM β-mercaptoethanol containing 0.014 to 1 mM L-[methyl-^14^C]carnitine and initial rates of transport were determined. As control, proteoliposomes not preloaded with unlabeled carnitine were used. The resulting data were plotted according to Michaelis-Menten, Lineweaver-Burk, and Eadie-Hofstee using the kinetic modules of the SigmaPlot software and GraphPad Prism.

### Pulse-chase experiments

*E. coli* WG170 cotransformed with pGP1-2 encoding T7 polymerase under the control of the *λ* pL promoter and the gene for the temperature sensitive *λ* repressor (cI857) under the control of the *lac* promoter^35^ and pT7-5/*caiT* were used. Cells were grown aerobically at 30 °C, harvested, washed three times with prewarmed M9 medium^38^ supplemented with 10 mM MgSO_4_ and trace elements. Cells were finally resuspended in M9 medium containing 10 mM MgSO_4_, trace elements, 0.5% glycerol, 0.001% thiamine, and all amino acids (0.05% each) except methionine and cysteine, and heat-induced expression of the *caiT* gene via the T7 promotor as well as pulse-chase experiments were performed as described^39^. Briefly, labeling was initiated by addition of 55 µCi [35S]methionine and the first sample was taken after 10 min (zero time point) followed by adding an excess of unlabeled methionine (2 mg/mL) and further samples were taken at given time points (5 min, 30 min, 120 min, overnight). Cells were disrupted by sonification, and membranes were prepared by ultracentrifugation as described^37^ and subjected to SDS-PAGE (12.5% gel). After drying the gel, radioactivity was detected using a storage Phosphor Screen and a Typhoon Trio Imager (Amersham Biosciences).

### Blue native gel electrophoresis

Blue native gel electrophoresis of purified CaiT variants was carried out as described previously^12,40^. Linear polyacrylamide (4–16.5%) gradient gels were cast with 4% overlay, and purified CaiT variants were loaded. The HMW Native Marker Kit (Amersham Biosciences) was used for molecular weight estimation under non-denaturing conditions.

### Analytical gel filtration

A Superdex 200 10/300 GL column (GE Healthcare) was used in combination with an ÄKTApurifier system (GE Healthcare). If necessary, a HiTrap Desalting column (GE Healthcare) was used for buffer exchange prior to measurement.

### Fluorescence measurements

Fluorescence was measured using a Fluoro-Max3 spectrofluorometer (Horiba Scientific). The excitation wavelength was set to 295 nm, and emission was recorded between 310 and 390 nm with slit widths of 2.5 nm. 80 μg of CaiT/mL were used to perform the measurements. Solubilized samples were analyzed in 100 mM potassium phosphate buffer, pH7.5, 2 mM β-mercaptoethanol, 10% glycerol, 200 mM imidazole, 0.04% *n*-dodecyl-*β*-D-maltoside at 20 °C. A HiTrap Desalting column (GE Healthcare) was used for buffer exchange of the eluted protein after its purification. Proteoliposomes (lipid to protein ratio 20:1) were analyzed in 100 mM potassium phosphate buffer, pH8, 2 mM β-mercaptoethanol, 5 mM MgCl_2_ at 25 °C. L-carnitine or γ-butyrobetaine (with neutralized pH) was stepwise added to final concentrations of 0 to 53 mM, and the cuvette was stirred for 5 min at 25 °C prior to measurement. All fluorescence data were corrected for a buffer blank and for dilution effects. The resulting data were plotted using the kinetic module of the SigmaPlot software After the fluorescence measurements of solubilized samples, gel filtration was applied to ensure that the protein did not aggregate during the measurement.

### Site-directed spin labeling and distance measurements

Membranes of *E. coli* BL21(DE3) pLysS transformed with pET21a/*caiT*(*ΔC*) were prepared and CaiT variants were purified and spin-labeled with 1 mM (1-oxyl-2,2,5,5-tetramethylpyrroline-3-methyl)-methanethiosulfonate (MTSSL, Toronto Research Chemicals) as described^41^. Purified, labeled CaiT was analyzed in 100 mM potassium phosphate buffer pH7.5, 2 mM β-mercaptoethanol, 0.04% (w/v) dodecyl β-D-maltoside). For reconstitution into liposomes, a lipid-to-protein (LPR) molar ratio of 40:1 (w/w) was adjusted.

Distance measurements were performed at Q band (~34.4 GHz) using conventional double electron-electron resonance (DEER) pulse sequence^42^. A commercial X/Q-band Elexsys E580 spectrometer (Bruker) power-upgraded to 200 W, a homebuilt Q-band spectrometer equipped with a 150 W amplifier as well as homebuilt TE102 rectangular resonators were used^43^. Aiming for a broadband excitation, lengths of all microwave (monochromatic, rectangular) pulses of the DEER sequence used were 12 ns, the pump frequency was set at the global maximum of the Q-band nitroxide EPR spectrum and the observer frequency was set 100 MHz lower. All DEER experiments were performed at 50 K. Protein solutions ready for measurement were filled into the 3 mm (outer diameter) quartz tubes, shock-frozen by immersion into liquid nitrogen and inserted into the pre-cooled resonator. To all samples, 10% *d*8-glycerol was added before freezing.

Acquired DEER traces were processed with the open-source DeerAnalysis package^44^ (version 2018), available at www.epr.ethz.ch/software.html. For background correction, 3D and 2D homogeneous distributions of spins were assumed for detergent-solubilized and liposome-reconstituted samples, respectively. Computation of distance distributions was stabilized by Tikhonov regularization characterized by the regularization parameter α using smoothness of the second derivative of *P(r)* as a regularization condition. Precision of distance extraction was estimated using the Validation tool of DeerAnalysis. An open source MMM package^20^, also available at www.epr.ethz.ch/software.html, was used to simulate relative populations of MTSSL rotamers, predict distance distributions and to compute tentative DEER responses (DEER form factors). Rotamer library R1A_298K_UFF_216_CASD was used.

### Protein Determination

Determination of protein was performed according to a modified Lowry method^45^ for total membrane protein, according to Bradford^46^ for detergent-solubilized protein, and by the Amido Black method^47^ for protein in proteoliposomes.

### Molecular Graphics and Analyses

Molecular graphics and analyses were performed with the UCSF Chimera package. Chimera is developed by the Resource for Biocomputing, Visualization, and Informatics at the University of California, San Francisco^48^.

## Supplementary information

Supporting Information

## Data Availability

The data that support the findings of this study are available from the corresponding authors upon reasonable request.
