# Supplementary material for: Comparison of the functional properties of trimeric and monomeric CaiT of Escherichia coli
Source: Sci Rep. 2019 Mar 7;9:3787. doi: 10.1038/s41598-019-40516-7 (PMC6406002; doi:10.1038/s41598-019-40516-7)
Supplement: Supplementary file 1 — Supporting Information [file 41598_2019_40516_MOESM1_ESM.pdf]

# Supporting Information

## Comparison of the functional properties of trimeric and monomeric CaiT of *Escherichia coli*

Susanne Bracher[‡], Daniel Hilger[‡$], Kamila Guérin[§#], Yevhen Polyhach[§], Gunnar Jeschke[§], Ralph Krafczyk[‡], Giacomo Giacomelli[‡], and Heinrich Jung[‡ 1]

[‡]From the Division of Microbiology, Department of Biology 1, Ludwig Maximilians University Munich, D-82152 Martinsried, Germany
[§]ETH Zurich, Laboratory of Physical. Chemistry, Vladimir-Prelog-Weg 2, Zurich, CH-8093, Switzerland
[#]present address: EPFL, Laboratory of Physics of Living Matter LPMV, Rte de la Sorge, Lausanne, CH-1015, Switzerland
[$]present address: School of Medicine, Department of Molecular and Cellular Physiology and Medicine, Stanford University, Stanford, CA 94304, USA

*

**Materials included:**

Table S1. Predicted energetic contribution of individual amino acids at the protomer interface to the binding free energy.

Figure S1. Alignment of sequences of members of the BCCT family.

Figure S2. Simulation of distance distributions between labeled sites in CaiT trimer with the rotamer library approach and computation of corresponding DEER form factors using the multi-spin system formalism.

Figure S3. Precision of distance distributions computed from the Q-band DEER data as estimated with the Validation Tool (DeerAnalysis).

Figure S4. Intensity normalized Q-band DEER time traces after background correction (DEER form factors).

Figure S5. Impact of given amino acid substitutions in CaiT on the growth stimulatory effect of L-carnitine.

Figure S6. Western blot analysis of CaiT with given amino acid replacements in *E. coli* JW0039 membranes.

Figure S7. The stability of CaiT and given variants in membranes of *E. coli.*

Supplementary References

**Table S1.** Predicted energetic contribution of individual amino acids at the protomer interface to the binding free energy. An *in silico* alanine scanning mutagenesis was performed using the Robetta server[1,2]. Calculations were performed on the structure of CaiT of *E. coli*[3]. The amino acids with the highest predicted contributions were listed. In addition, T304 was included since the corresponding amino acid (T351) was previously shown to contribute to destabilization of the BetP trimer[4]. Amino acids replaced in this investigation were highlighted in bold.

| **Amino acid** | **Location** | $\Delta\Delta G_{bind}$ [kcal mol$^{-1}$] |
|---|---|---|
| W53 | periplasmic end TMD 2 | 1.78 |
| W57 | periplasmic end TMD 2 | 1.86 |
| E132 | loop 3 connecting TMDs 3 and 4 | 1.23 |
| T287 | helix 7 | 1.42 |
| **D288** | helix 7 | 2.23 |
| **M295** | helix 7 | 1.22 |
| **R299** | helix 7 | 3.58 |
| F302 | helix 7 | 2.00 |
| **T304** | helix 7 | 0.44 |
| I307 | loop 7 connecting helix 7 with TMD8 | 2.06 |

```
                         TMD1                                   TMD2
EcCaiT   9 GIEPKVFFPPLIIVGILCWLTVRDLDAANVVINAVFSYVTNVWGWAFEWYMVVMLFGWFW 68
PmCaiT   9 GIEPKVFFPPLIIVGILCWLTVRDLDASNEVINAVFSYVTNVWGWAFEWYMVIMFGGWFW 68
CgEctP  20 KSDPFIFSISVGFIVVFVIATIALGEKARTTFSAIAGWLLENLGWMYIGGVSLVFIFLMG 79
PsBetP  13 RMNAPVFYFAASFILIFGIVVIAFPQASGEWLLAAQNWAANTVGWYYMMVMTLYLVFVVV 72
EcBetT  12 KINPVVFYTSAGLILLFSLTTILFRDFSALWIGRTLDWVSKTFGWYYLLAATLYIVFVVC 71
CgBetP  57 SLNWSVIVPALVIVLATVVWGIGFKDSFTNFASSALSAVVDNLGWAFILFGTVFVFFIVV 116
SmBetS  52 KVNLPVFVGSVAVIALFVGIGVIAPKRAESIFSGMQTAILSGFGWLYLLSVAVFLFSMLF 111
BsOpuD   3 KHISSVFWIVIAITAAAVLWGVISPDSLQNVSQSAQAFITDSFGWYYLLVVSLFVGFCLF 62
                ::     .        :   .              .  ** :     : .   .

                              TMD7                         Helix7
EcCaiT 245 ICVACGLQKGVRIASDVRSYLSFLMLGWVFIVSGASFIMNYFTDSVGMLLMYLPRMLFYT 304
PmCaiT 245 ICVAFGLQKGVKIASDVRTYLSFLMLGWVFIVGGASFIVNYFTDSVGTLLMYMPRMLFYT 304
CgEctP 257 ISVASGLDKGIKLLSNINIAMAVALMFFILFTGPTLTLLRFLVESFGIYASWMPNLMFWT 316
PsBetP 248 LVAIAGVDKGVRVMSDINMLLACALLLFVLFAGPTQHLLNTLVQNIGDYLGALPSKSFDV 307
EcBetT 247 ISVTSGVDKGIRVLSELNVALALGLILFVLFMGDTSFLLNALVLNVGDYVNRFMGMTLNS 306
CgBetP 292 FSAISGVGKGIQYLSNANMVLAALLAIFVFVVGPTVSILNLLPGSIGNYLSNFFQMAGRT 351
SmBetS 287 ISVVTGVEKGVRILSETNLFLAVLLMLFVLVVGPTGTLMRDFVQNIGLYLDSLVLRTFNI 346
BsOpuD 239 LSAWSGLGKGIKYLSNTNMVLAGLLMLFMLVVGPTVLIMNSFTDSIGQYIQNIVQMSFRL 298
           : .  *: **::  *: .  ::  :  :::. . :  ::. :  ..*     :

                                  TMD8                        TMD9
EcCaiT 305 -DPI--AKGGFPQGWTVFYWAWWVIYAIQMSIFLARISRGRTVRELCFGMVLGLTASTWI 361
PmCaiT 305 -DPI--GKGGFPQAWTVFYWAWWVIYAIQMSIFLARISKGRTVRELCLGMVSGLTAGTWL 361
CgEctP 317 -DSFQ-DNPGWQGKWTVFYWAWTICWSPYVGMFVARISRGRTVREFIGGVLALPAIFGVV 374
PsBetP 308 -YAYN-EPSDWLGGWTVFYWAWWIAWAPFVGLFIARISRGRTIREFVFGVLLIPLGFTLA 365
EcBetT 352 -FAFD-RPVEWMNNWTLFFWAWWVAWSPFVGLFLARISRGRTIRQFVLGTLIIPFTFTLL 364
CgBetP 352 AMSADGTAGEWLGSWTIFYWAWWISWSPFVGMFLARISRGRSIREFILGVLLVPAGVSTV 411
SmBetS 347 -YAY--EPRPWIDSWTLFYWAWWISWSPFVGMFIARISRGRTVREFVTAVLFVPAMFTFL 403
BsOpuD 299 -TPNDPEKREWINSWTIFYWAWWISWSPFVGIFIARVSRGRTIREFLIGVLVTPCILTFL 357
                     :   **:*:*** : ::  :.:*:**:*:**::*::  . :
```

**Figure S1.** Alignment of sequences of members of the BCCT family. The alignment of the complete amino acid sequences was performed with CLUSTAL Omega[5]. Shown are the regions around TMDs 1 to 2 and TMDs 7 to 9. Amino acids involved in inter-protomer interactions based on the crystal structures of BetP[4] and CaiT[3] are colored. Interacting sites have the same color. Sites experimentally shown to be important for trimer stability [BetP[4]; CaiT, this investigation] are in bold. The amino acid proposed to be cause of the substrate-induced change in tryptophan fluorescence (W323)[3] is highlighted in white on black background. EcCaiT, CaiT of *Escherichia coli* (P31553); PmCaiT, CaiT of *Proteus mirabilis* (B4EY22); CgEctP, EctP of *Corynebacterium glutamicum* (Q79VE0); PsBetT, BetT of *Pseudomonas syringae* (Q4ZLW8); EcBetT, BetT of *E. coli* (P0ABC9); CgBetP, BetP of *C. glutamicum* (P54582); SmBetS, BetS of *Sinorhizobium meliloti* (Q92WM0); BsOpuD, OpuD of *Bacillus subtilis* (P54417).

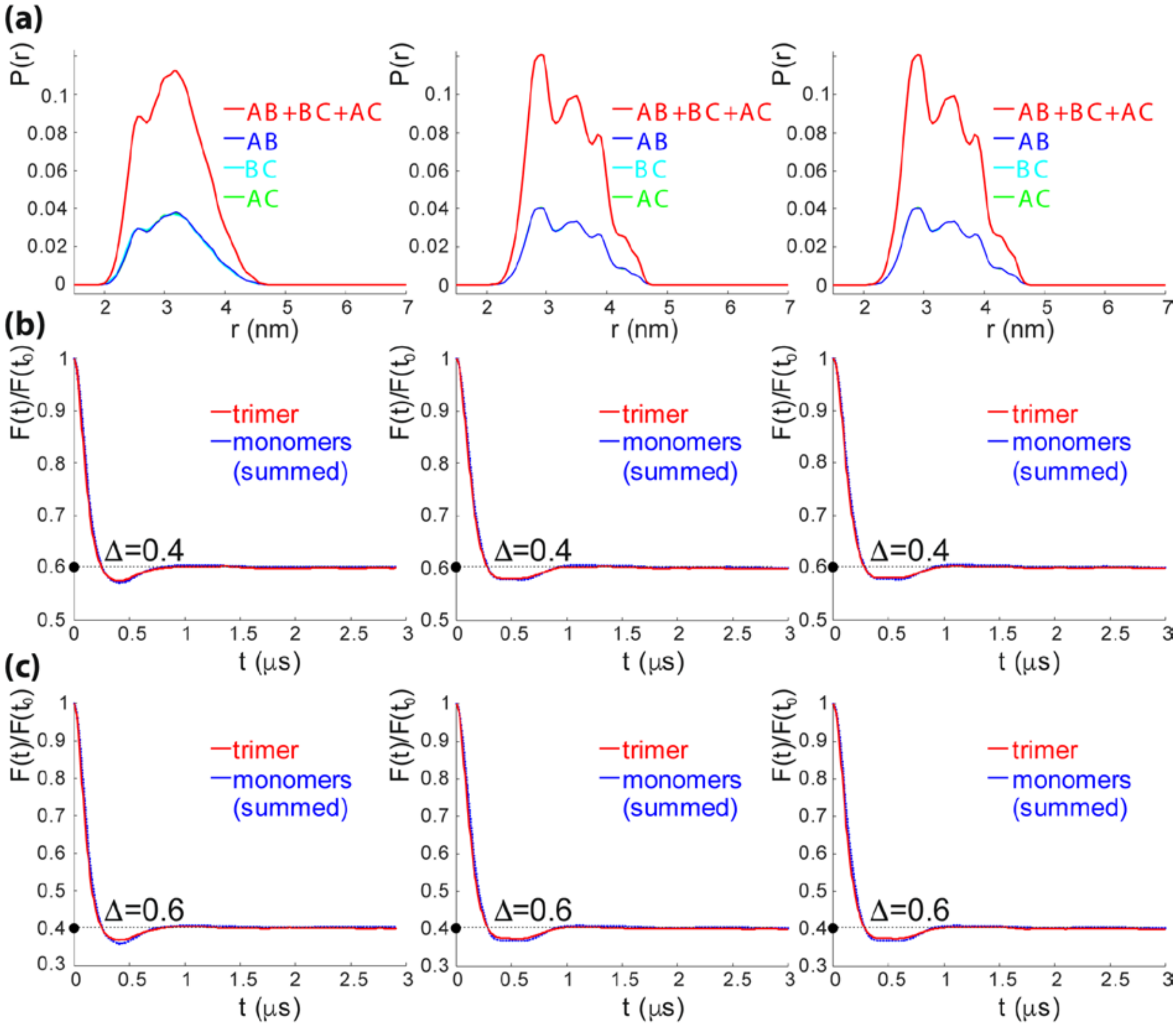


**Figure S2.** Simulation of distance distributions between labeled sites in CaiT trimer with the rotamer library approach[6] and computation of corresponding DEER form factors using the multi-spin system formalism[7,8]. **(a)** Interspin distance distributions in CaiT trimer computed using the following pdb template structures: 2WSX (left), 3HFX (middle) and 3HFX – carnitine removed (right). Distance distributions corresponding to a trimer (red curves) were obtained by summing up all (three) pairwise distance distributions (blue, cyan and green curves). **(b)** Simulated DEER form factors corresponding to a trimer (i.e. between all three labeled sites, red curves) in comparison to summed form factors stemming from pairwise distance distributions. Computed for a total modulation depth of $\Delta = 0.4$. Differences between the two form factors are negligible. **(c)** The same as in (b) computed for a total modulation depth of $\Delta = 0.6$. Differences between the two form factors increased and are well seen. Rotamer library R1A_298K_UFF_216_CASD as implemented into an open-source package MMM (version 2018.1) was used for all simulations.

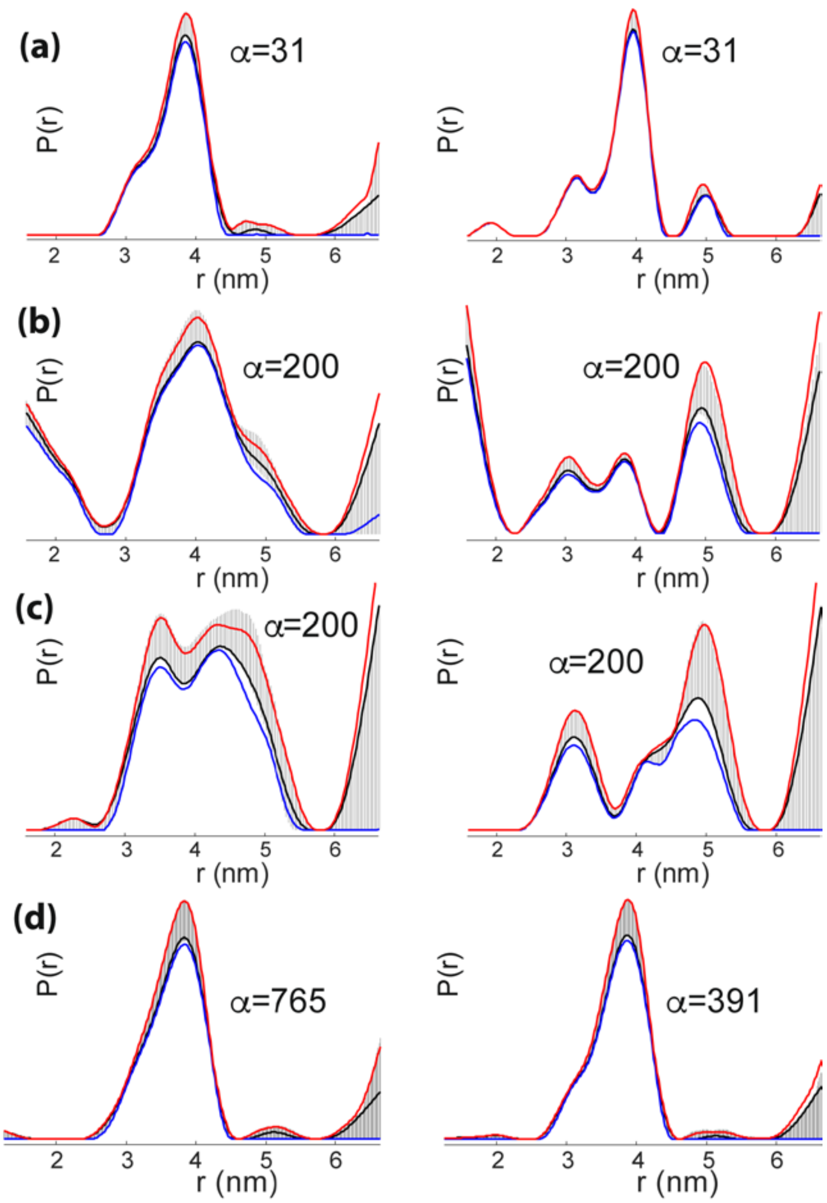


**Figure S3.** Precision of distance distributions computed from the Q-band DEER data as estimated with the Validation Tool (DeerAnalysis). The analysis was performed using primary DEER data for respective mutant shown in Figs. 4 and 5. During the validation procedure, distance extraction with chosen initial number of background decay models (trials) was performed for each case (given below). For more realistic error estimation, ensembles of trials were pruned to keep only those models whose r.m.s.d. was lying within 1.15*r.m.s.d.$_{min}$ (default value). In every panel: grey-shaded areas indicate the full variation of the probability of a given distance over all trials; solid black line corresponds to a distance distribution for which the background fit has the lowest r.m.s.d.; red and blue lines denote a lower and an upper error estimates (the mean value plus/minus two times the standard deviation) for a given distance respectively. **(a)**. CaiT(ΔC)-R1$_{118}$ trimer in proteoliposomes (left, 2160 trials, primary data are in Fig. 4a) and detergent (right, 4312 trials). **(b)**. CaiT(ΔC)-R$_{118}$/D288A mutant in proteoliposomes (left, 2340 trials) and detergent (right, 3960 trials). **(c)**. CaiT(ΔC)-R$_{118}$/D288R mutant in proteoliposomes (left, 4212 trials) and detergent (right, 3630 trials). **(d)**. CaiT(ΔC)-R1$_{118}$ trimer in proteoliposomes at the absence (left, 4368 trials) and the presence of carnitine (right, 4368 trials), primary data are in Fig. 4c.

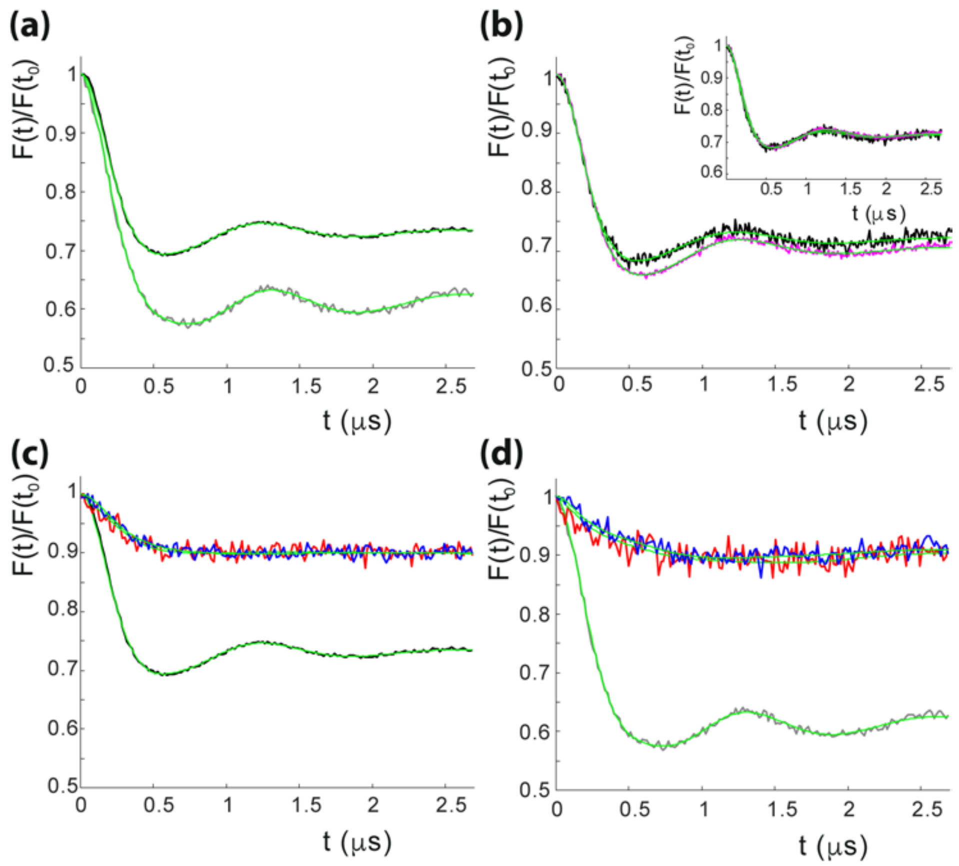


**Figure S4.** Intensity normalized Q-band DEER time traces after background correction (DEER form factors). **(a)** CaiT(ΔC)-R$1_{118}$ trimer reconstituted into proteoliposomes (black) and detergent solubilized (grey). **(b)** CaiT(ΔC)-R$1_{118}$ trimer reconstituted into proteoliposomes prepared at the presence (magenta) and absence (black) of carnitine. Inset shows form-factors scaled to the same modulation depth for better direct comparison. **(c)** CaiT(ΔC)-$R_{118}$/D288A (red) and CaiT(ΔC)-$R_{118}$/D288R (blue) mutants in comparison to CaiT(ΔC)-R$1_{118}$ trimer – all reconstituted into proteoliposomes. **(d)** CaiT(ΔC)-$R_{118}$/D288A (red) and CaiT(ΔC)-$R_{118}$/D288R (blue) mutants in comparison to CaiT(ΔC)-R$1_{118}$ trimer – all detergent-solubilized. Green traces throughout represent fits to experimental DEER form factors.

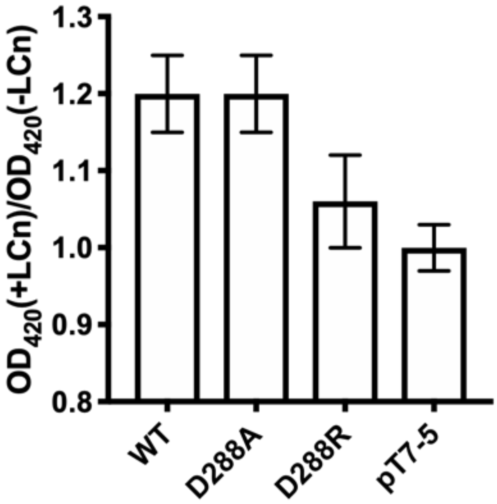


**Figure S5.** Impact of given amino acid substitutions in CaiT on the growth stimulatory effect of L-carnitine. *E. coli* JW0039 (*ΔcaiT*) was transformed with plasmid pT7-5/*caiT*(wild-type), pT7-5/*caiT*-D288A, pT7-5/*caiT*-D288R, or pT7-5 without *caiT*. Cells were grown in LB medium supplemented with 100 µg/mL ampicillin under anaerobic conditions (15 mL glass tubes completely filled with medium and tightly sealed) at 37°C. Growth was analyzed in the presence and absence of 50 mM L-carnitine by measuring the optical density at 420 nm ($OD_{420}$). Starting at an $OD_{420}$ of 0.03, cells were cultivated for 6 h (middle of exponential growth phase). The ratio of $OD_{420(6h)}$(+L-carnitine)/ $OD_{420(6h)}$(-L-carnitine) was taken as measure of the growth stimulating effect of L-carnitine. Error bars represent standard deviations calculated from four biological repeats of the experiment.

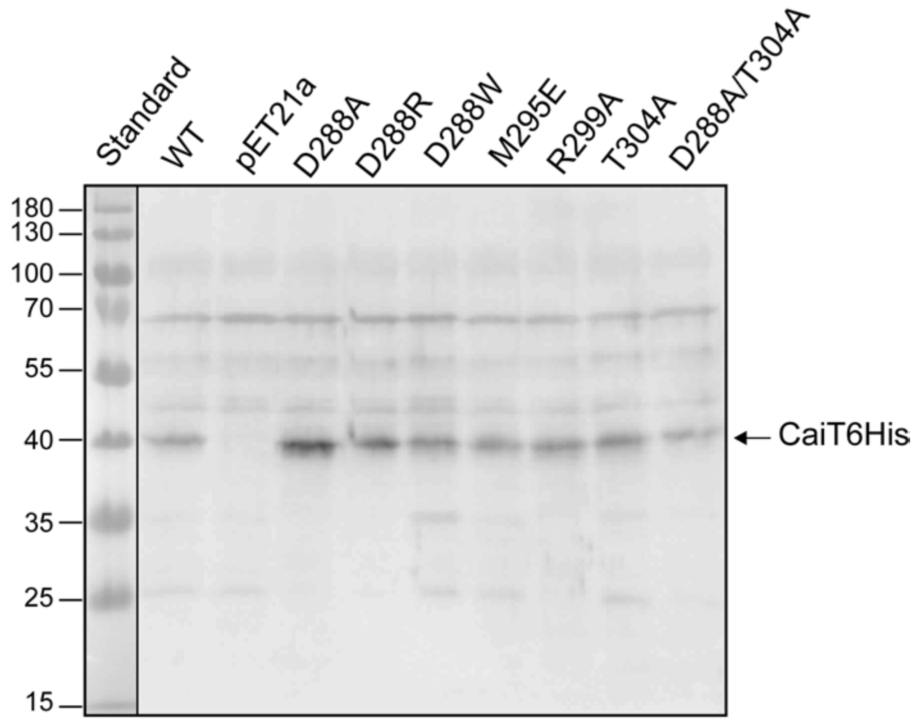


**Figure S6.** Western blot analysis of CaiT with given amino acid replacements in *E. coli* JW0039 membranes. HRP-linked mouse anti-His IgG was used to detect CaiT. PageRuler™ Prestained Protein Ladder was used for molecular size estimation. The figure is a composite of two images of one blot recorded with the Fusion FX imaging system (Vilber): a white light image to detect the prestained protein standard, and a luminescence image. Relative molecular masses are indicated in kDa. The blot corresponds to Fig. 6a.

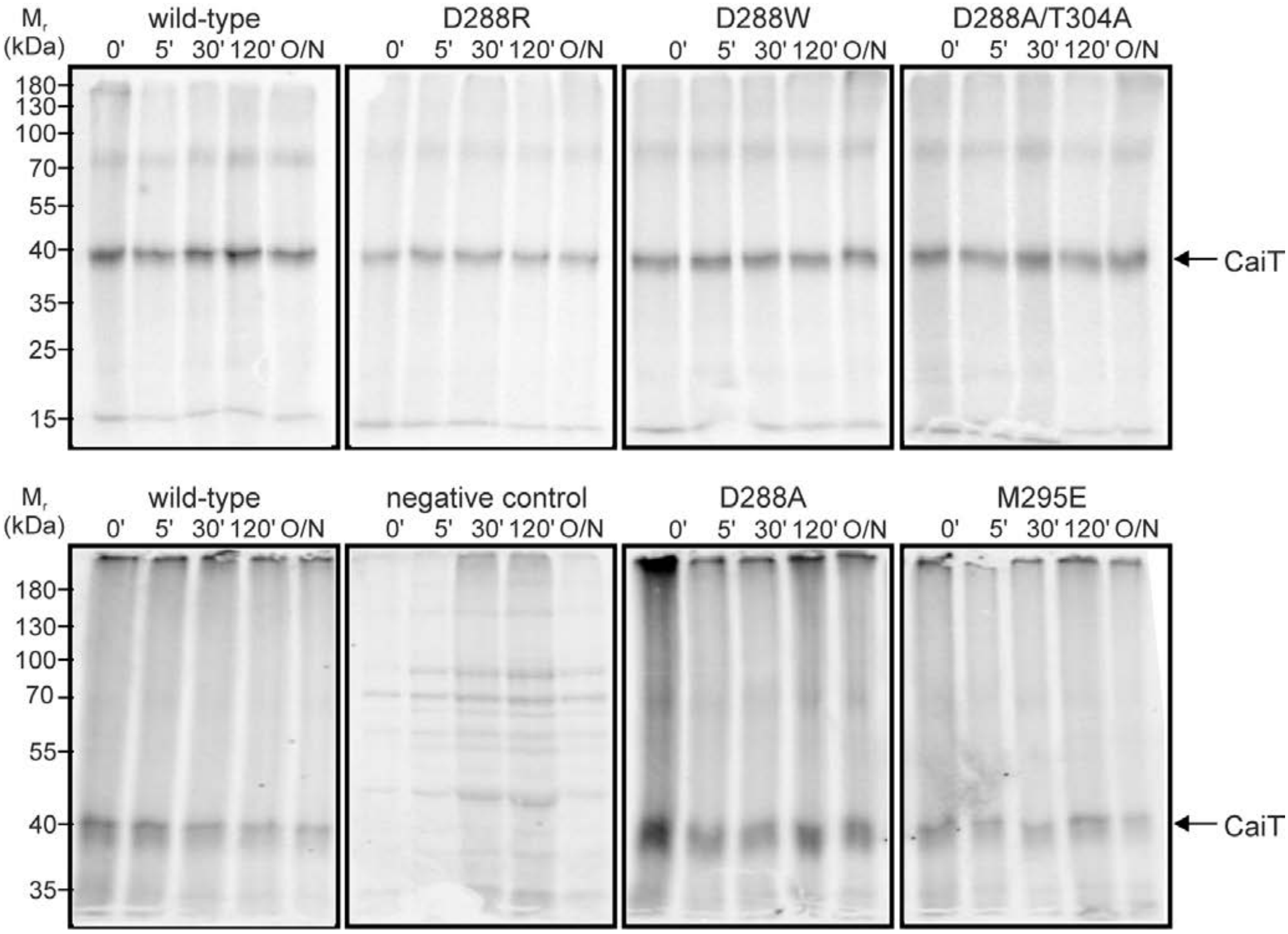


**Figure S7.** The stability of CaiT and given variants in membranes of *E. coli.* The stability was tested in a pulse-chase experiment. For this purpose cells were incubated with [$^{35}$S]methionine at 30°C for 10 min before removal of the zero-time point aliquot and addition of an excess of unlabeled methionine (2 mg/mL final concentration). Further aliquots were taken at given time points of incubation. Cells were disrupted by sonication, membranes were prepared and subjected to SDS-PAGE (12.5%). Radioactivity was detected with a Phosphor imager after drying the gel. The gels corresponds to Fig. 9c.

**Supplementary References**


1 Kortemme, T., Kim, D. E. & Baker, D. Computational alanine scanning of protein-protein interfaces. *Sci. STKE* **2004**, pl2 (2004).

2 Kortemme, T. & Baker, D. A simple physical model for binding energy hot spots in protein-protein complexes. *Proc. Nat. Acad. Sci. U.S.A.* **99**, 14116-14121 (2002).

3 Schulze, S., Koster, S., Geldmacher, U., Terwisscha van Scheltinga, A. C. & Kühlbrandt, W. Structural basis of $Na^+$-independent and cooperative substrate/product antiport in CaiT. *Nature* **467**, 233-236 (2010).

4 Perez, C., Khafizov, K., Forrest, L. R., Kramer, R. & Ziegler, C. The role of trimerization in the osmoregulated betaine transporter BetP. *EMBO Rep.* **12**, 804-810 (2011).

5 Sievers, F. *et al.* Fast, scalable generation of high-quality protein multiple sequence alignments using Clustal Omega. *Mol. Syst. Biol.* **7**, 539 (2011).

6 Polyhach, Y., Bordignon, E. & Jeschke, G. Rotamer libraries of spin labelled cysteines for protein studies. *Phys. Chem. Chem. Phys.* **13**, 2356-2366 (2011).

7 Jeschke, G., Sajid, M., Schulte, M. & Godt, A. Three-spin correlations in double electron-electron resonance. *Phys. Chem. Chem. Phys.* **11**, 6580-6591 (2009).

8 von Hagens, T., Polyhach, Y., Sajid, M., Godt, A. & Jeschke, G. Suppression of ghost distances in multiple-spin double electron-electron resonance. *Phys. Chem. Chem. Phys.* **15**, 5854-5866 (2013).
